# *Calendula arvensis* (Vaill.) L.: A Systematic Plant Analysis of the Polar Extracts from Its Organs by UHPLC-HRMS

**DOI:** 10.3390/foods11030247

**Published:** 2022-01-18

**Authors:** Marika Fiorentino, Claudia Gravina, Simona Piccolella, Maria Tommasina Pecoraro, Marialuisa Formato, Adriano Stinca, Severina Pacifico, Assunta Esposito

**Affiliations:** Department of Environmental Biological and Pharmaceutical Sciences and Technologies, University of Campania “Luigi Vanvitelli”, Via Vivaldi 43, 81100 Caserta, Italy; marika.fiorentino@unicampania.it (M.F.); claudia.gravina@unicampania.it (C.G.); simona.piccolella@unicampania.it (S.P.); mariatommasina.pecoraro@unicampania.it (M.T.P.); marialuisa.formato@unicampania.it (M.F.); adriano.stinca@unicampania.it (A.S.); assunta.esposito@unicampania.it (A.E.)

**Keywords:** *Calendula arvensis*, Asteraceae, UHPLC-Q*q*TOF-MS/MS analysis, flavonoids, triterpene saponins, traditional food, antioxidants

## Abstract

*Calendula arvensis* (Vaill.) L. (field marigold, Asteraceae) is an alimurgic plant, whose flowers and leaves are a common part of local food dishes. The diversity in polar specialized metabolites is herein unraveled, with the aim to further promote and valorize the food use of the plant. To this purpose, following the plant dissection of its organs (florets, fruits, leaves, bracts, stems, and roots), ultrasound assisted maceration has been employed in order to recover phenols and polyphenols. Through an untargeted UHPLC-HR MS (Ultra-High-Performance Liquid Chromatography-High-Resolution Mass Spectrometry) approach, and deeper investigation of the fragmentation patterns of each compound by tandem mass spectrometry, the florets’ constitution in triterpene saponins and flavonol glycosides has been highlighted, whereas hydroxycinnamoyl compounds are mainly in bracts and fruits. The antiradical and reducing capabilities of the organs’ extracts have been assessed, and data acquired have been analyzed by cluster analysis, which allowed bracts and fruits to be observed, despite their negligible food use, as the most active extracts. Chemical and antioxidant data on the diverse organs of field marigold suggest new investigative food and nutraceutical scenarios of this plant, also revalorizing and preserving its traditional uses.

## 1. Introduction

Wild edible plants (WEPs), described as native species naturally growing, have been among the main food ingredients of rural communities linking human life since ancient times [1,2]. In the Mediterranean area, the traditional uses of WEPs were introduced in ancestors’ diets for their curative properties, and nowadays, they continue to represent a considerable part of the Mediterranean Diet. WEPs as basic food stuffs in many local folk cuisines [3,4,5,6,7]. The renewed interest in WEPs is largely due to the growing knowledge of the healthy role of phytochemical compounds, so much so that WEPs can be defined as “functional foods”, being a good source of bioactive molecules and dietary supplements [8,9,10]. This is especially true in countries where the native vascular flora is particularly rich, such as Italy [11].

Indeed, the increased interest in ethnobotanical studies of local wild plants [7,12] emerged out of a need to create new products for the food industry with beneficial properties, as well as for sustainable agriculture because of the low impact of their cultivation on the environment [2,13,14,15,16]. In this scenario, knowledge of edible plants biodiversity available in the local food tradition, and of new pathways for the eco-sustainable enhancement of food resources, constitute crucial aspects to intercept in marginal or valuable areas (e.g., Nature Reserves and National Parks), in full agreement with the EU’s biodiversity strategy plan for 2030 [17]. From the ethnobotanical point of view, Asteraceae is one of the most studied angiosperm families [18,19], thanks to their great diversity and wide worldwide distribution [20]. Within this family, the genus *Calendula* L. currently includes 15 species and several infraspecific taxa [21]; it is the only genus of the tribe Calenduleae in the Mediterranean region, spreading from Macaronesia to South Western (SW) Asia [21].

In the folk tradition, plants of genus *Calendula* have a long history, being widely used as a curative remedy against several diseases as well as an ingredient in local dishes [22]. Within this genus, *Calendula arvensis* (Vaill.) L. (known as field marigold) is an herbaceous annual species widely distributed in Europe, northern Africa, SW Asia, and the Macaronesian region, and also naturalized in other temperate regions [23,24]. *C. arvensis* is a monoecious, self-compatible, and polymorphic annual weed. It can be found everywhere, being able to grow in cultivated fields, along roadsides, and in disturbed sites on different soil types [25,26]. It has lance-shaped leaves with secretory trichomes, hairy stems, small capitula (2–3 cm) with yellow ligulate ray florets, and tubulous central florets; the fruits are achenes of three different shapes, the outers are rostrate and cymbiform, while the sub-peripherals are annular. The flowering period ranges from November to May [23,27,28]. It is reported that its rostrate and cymbiform achenes are larger and heavier than annular achenes [23]. The adaption to long-range dispersal also distinguish them from annular achenes. Moreover, achenes germinate across a wide temperature range (in light and in darkness). Rostrate and cymbiform achenes produce seedlings best able to emerge from deeper burial depths, and are able to exhibit earlier flowering than seedlings from annular achenes; fruiting also takes place without pollinators. It is a prolific seed producer, forming a persistent seed bank. All these features favor the species establishment and expansion in unpredictable and disturbed habitats [23].

*C. arvensis* has a long tradition of uses in different Italian regions, where it is utilized as an edible alimurgic plant. In particular, among the diverse plant organs, the flower and leaves are frequently consumed. Literature reports the use of field marigold flower as a boiling vegetable, whereas the leaves are key ingredients in cooking a typical soup [7,29]. Indeed, *C. arvensis* has a dual value. In fact, beyond its use as foodstuff, its role as phytomedicine has been broadly employed [30]. *C. arvensis*-based decoction are used for treating wounds, contusion, and burns; whereas field marigold tea is known for its antiseptic and astringent properties. Flower-based preparations are suggested for external use to preserve skin firmness, or to counteract skin inflammation, and to regenerate damaged tissues [31,32,33]. The flower extracts have been also extensively investigated for their antioxidant efficacy and anticandidal, antifungal, and antimicrobial activities [34], whereas the cytotoxic effects against human myeloid cells [34] and breast cancer lines [35] emphasize that the potential benefits for humans from this wild plant could be multidimensional.

Indeed, despite of numerous data on *C. arvensis* bioactivity, few studies combine it with a deeper investigation of the chemical composition of the plant organ utilized for extraction purposes [30,36,37]. Furthermore, an inappropriate indication of the part used to study biological activity is common in the literature, so much so that the aerial parts are often cited as the investigated plant matrix, without taking care in the botanical characterization of the aerial part that consists of more organs. It is certain that while there are diverse compositive data for field marigold essential oils [30,36,37], few and fragmentary information are reported in relation to phenol compounds [38,39], which are mainly attentive for their benefits to humans. Indeed, considering that the biosynthesis and following accumulation of polyphenols and other specialized metabolites differ in the various plant organs [40,41,42], in order to achieve a detailed investigation of local *C. arvensis* to gain insight into the chemical constituents and to promote the sustainable food use of its different organs, plant dissection is herein proposed as preliminary to extraction. The latter was carried out in order to strengthen the recovery of phenols and polyphenols from the six *C. arvensis* organs, such as florets, fruits, leaves, bracts, stems, and roots. UHPLC-HRMS (Ultra-High-Performance Liquid Chromatography-High-Resolution Mass Spectrometry) techniques, in an untargeted approach, were exploited to unravel the chemical complexity of different organs of *C. arvensis,* as well as antioxidant assessment through three different assays.

## 2. Materials and Methods

### 2.1. Plant Material Collection, Organ Separation, and Extraction

**Figure 1:**
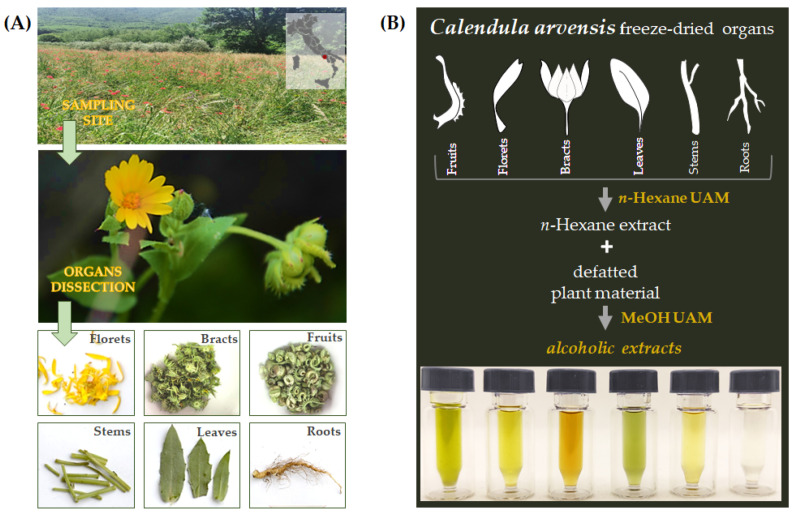
*C. arvensis* sampling site and organs separation (**A**); fractionation scheme of the plant’s freeze-dried organs (**B**).

*Calendula arvensis* plants were collected in May 2021 in southern Italy in the municipality of Roccaromana (Caserta, Italy; 41°16′30.36″ N 14°13′19.92″ E, 163 m a.s.l.) (Figure 1A). Taxonomic identification was performed following [28,29]. A voucher specimen (CE0131) has been deposited in the Herbarium of the Department of Environmental, Biological, and Pharmaceutical Sciences and Technologies of the University of Campania Luigi Vanvitelli (Caserta, Italy). Sampled plants came from an uncultivated land colonized mainly by *Papaver rhoeas* L. subsp. *rhoeas* and other ruderal species. As per the Bioclimatic map of Europe [43], this territory is located in the transition zone between the Pluvioseasonal Oceanic Mediterranean and Temperate Oceanic Submediterranean bioclimates. From the pedological point of view, soil is a Luvi-Vitric Andosol and is ascribable to the great land system of the “foothills plain of limestone reliefs” [44]. Immediately after harvesting, each plant material was dissected by hand into fruits, ligulate florets (henceforth referred to as florets), receptacle with involucral bracts (throughout the text, indicated as bracts), leaves, stems, and roots, then labeled, and stored in liquid nitrogen (Figure 1A).

Each plant organ was first lyophilized and pulverized by a rotating knives homogenizer. Dried material underwent ultrasound assisted maceration (UAM; Branson Ultrasonics^TM^ Bransonic^TM^ M3800-E; Danbury, CT, USA) using first *n*-hexane and then methanol as extractive solvents. The drug/solvent ratio was 1:20 (g drug: mL solvent); three UAM cycles by each solvent were carried out (30 min for each; Figure 1B). The alcoholic extracts were chemically analyzed through UHPLC-ESI-Q*q*TOF-MS/MS analysis, and their antioxidant capability was assessed. 

### 2.2. UHPLC-ESI-QqTOF-MS and MS/MS Analyses

The alcoholic extracts from *C. arvensis* organs were profiled by a NEXERA UHPLC system (Shimadzu; Tokyo, Japan) equipped with Luna^®^ Omega C-18 columns. A linear gradient was applied for separative purposes with water (A) and acetonitrile (B), both with 0.1% formic acid: held at 5%, for 1 min; 1–7 min, 5–17.5% B; 7–9 min, 17.5–25% B; 9–18 min, 25–55% B; 18–20 min, 55–95% B. The mobile phase composition was maintained at 95% B for another 1 min. Thus, the starting conditions were restored in 1 min, while system re-equilibration was in 2 min. The flow rate was 0.5 mL/min. The injection volume was 2.0 μL. MS analysis was achieved by the AB SCIEX Triple TOF^®^ 4600 (AB Sciex; Concord, ON, Canada), equipped with a DuoSpray^TM^ ion source, which operated in the negative ESI mode. 

The Q*q*TOF HRMS method consisted of a full scan TOF survey (accumulation time 249.9 ms, 100–1500 Da) and eight IDA (information-dependent acquisition). The MS parameters were as follows: curtain gas 35 psi, nebulizer gas 60 psi, heated gas 60 psi, ion spray voltage 4.5 kV, and interface heater temperature 500 °C. The instrument was controlled by Analyst^®^ TF 1.7 software, while data processing was through PeakView^®^ software version 2.2. 

The TOF-MS/MS parameters for the analysis of flavonoids and hydroxycinnamic and hydroxybenzoic acids were −100 V of declustering potential (DP), −40 V of Collision energy (CE), and −15 V of Collision energy spread (CES). For the characterization of triterpenoid saponins, the parameters were as follows: DP −120 V, CE −100 V, and CES −25 V.

### 2.3. Antioxidant Assessment

The alcoholic extracts from *C. arvensis* organs were tested at 100, 50, 25, 10, and 2.5 µg/mL towards the ABTS [2,2′-azinobis-(3-ethylbenzothiazolin-6-sulfonic acid)] radical cation and 2,2-diphenyl-1-picrylhydrazyl (DPPH) radical. The ABTS radical cation was prepared as in Pacifico et al. [45]. After the ABTS^•+^ solution was diluited in Phosphate-buffered saline (PBS; pH 7.4) to achieve an absorbance of 0.7, that was recorded at 734 nm. All organ alcoholic extracts were dissolved in the ABTS^•+^ solution in order to achieve the final tested dose level, and the absorbance values were taken after 6 min by a Victor3 spectrophotometer (Perkin Elmer/Wallac; Waltham, MA, USA). A blank, in which the organ extracts were replaced with solvents, was also prepared. 

The DPPH free radical scavenging capacity was also evaluated as previously described [45]. The Victor3 spectrophotometer (Perkin Elmer/Wallac; Waltham, MA, USA) was employed for recording the absorbance at 517 nm. A blank, in which methanolic extracts were replaced with solvents, was used as a reference. In both the antiradical assays, Trolox (4, 8, 16, 32 µM) was the positive standard. Three replicate measurements for each samples (three for each concentration) were performed. 

The potassium ferricyanide reducing power (PFRAP) assay was also performed to estimate the reducing power of the investigated alcoholic extracts (at 100, 50, 25, 10, and 2.5 µg/mL final concentration levels). The absorbance was measured at 700 nm [46,47]. A blank was considered, preparing a solution with PFRAP reagent without samples, as well as Trolox as a positive standard. 

All data were expressed as the mean ± standard deviation (SD).

### 2.4. Statistical Analysis

A multivariate analysis approach by ClustVis (https://biit.cs.ut.ee/clustvis/, accessed on 20 October 2021) was adopted to explore and clarify quali-quantitative compositive data compounds in each organ.

Numerical clustering of antioxidant assay (DPPH, ABTS, and PFRAP) data was made on the basis of mean values of three replicates for each of five extract concentrations tested for each of the six *C. arvensis* organs (fruits, florets, bracts, stems, leaves, and roots), using the SYN-TAX software [48].

## 3. Results and Discussion

### 3.1. UHPLC-QqTOF-MS/MS Analysis of Calendula arvensis (Vaill.) L.

**Figure 2:**
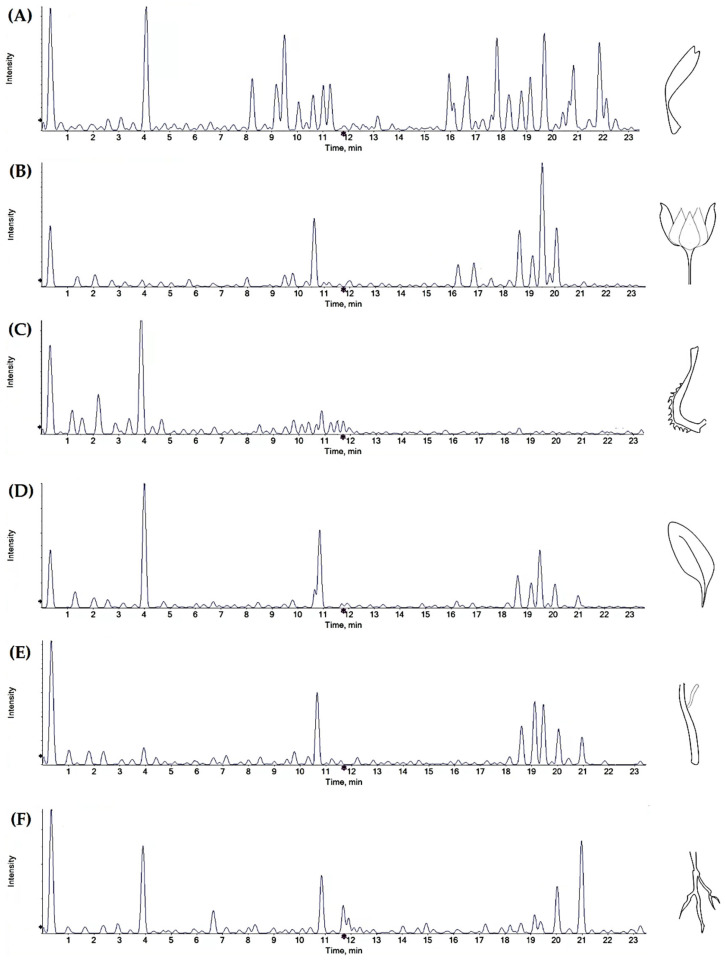
TICs (Total Ion Chromatograms) of the alcoholic extracts from (**A**) florets, (**B**) bracts, (**C**) fruits, (**D**) leaves, (**E**) stems, and (**F**) roots of *Calendula arvensis*.

To investigate the chemical composition, mainly in terms of polyphenols, of the diverse organs (florets, fruits, bracts, leaves, stems, and roots) of *C. arvensis*, the cryo-dried plant materials underwent ultrasound assisted maceration first in *n*-hexane, to remove more lipophilic components (e.g., fatty acids). Thus, the defatted plant matrices were then extracted with methanol. The alcoholic extracts obtained, were analysed by means of UHPLC-Q*q*TOF-MS/MS analysis, taking into account the chemical features of the compounds. The Total Ion Chromatograms (TICs; Figure 2) highlight that hydroxycinnamoyl-based compounds, flavonoids, and triterpenoid saponins diversely occurred in the different organs. Different TOF-MS/MS parameters (including collision energy and declustering potential) were utilized in order to reach a comprehensive fragmentation for each identified class of compounds, thus putatively unravelling the compounds’ MS/MS chemical features. TOF-MS and TOF-MS/MS data of all the compounds are listed in Table 1.

#### 3.1.1. Hydroxycinnamic Acids Derivatives 

Compounds **6**–**9**, **23**, **30**, **31,** and **33** are hydroxycinnamoyl derivatives. In particular, compound **6** with the [M−H]^−^ ion at *m*/*z* 341.0880 was tentatively identified as caffeoyl hexose based on its TOF-MS/MS spectrum, which displayed the ion at *m*/*z* 251.0570, according to the hexose cross-ring cleavage with the neutral loss of 90 Da, and the ion at *m*/*z* 179.0354, due to the loss of 162.05 Da (hexose-H_2_O). The dehydration of the ion at *m*/*z* 179.0354 provided the base peak of the other characteristic caffeic acid ion at *m*/*z* 161.0252. Otherwise, the caffeate decarboxylation produced the fragment ion at *m*/*z* 135.0456. This compound was previously identified in the aerial part of *C. arvensis* L. [39]. Compounds **7**, **8**, **9**, and **23** belong to the chlorogenic acid family. The TOF-MS/MS spectra of the first two metabolites, with relative deprotonated molecular ions at *m*/*z* 353.0877 and 353.0876, were in agreement with two geometric isomers of 5-*O*-caffeoylquinic acid (C_16_H_18_O_9_). In fact, TOF-MS/MS fragment ions were in the quinate, which appeared as the base peak at *m*/*z* 191.0561 and 191.0566, respectively [49]. Compound **9** with the [M−H]^−^ ion at *m*/*z* 367.1038 was likely 5-*O*-feruloyl quinic acid. To strengthen this hypothesis, the TOF-MS/MS fragment ions at *m*/*z* 193.0513 (ferulate) and 191.0560 were observed. The compound **23** with [M−H]^−^ ion at *m*/*z* 515.1197 and fragment ions at *m*/*z* 353.0878, 191.0561 (base peak), and 179.0346 was tentatively identified as 3,5-di-*O*-caffeoylquinic acid, which was reported as constituent of *C. officinalis* inflorescences, as well as of *C. arvensis* aerial parts [39]. Compounds **30**, **31**, and **33**, with relative [M−H]^−^ ion at *m*/*z* 695.1270, 695.1267, and 695.1268, were tentatively identified as tricaffeoylcitric acid isomers. TOF-MS/MS experiments, whose spectra are in Figure S1, displayed the sequential loss of three dehydrated caffeoyl moieties to generate the fragment ions at *m*/*z* 533.09, 371.06, and 209.02. The fragment ion at *m*/*z* 371.06 underwent further H_2_O loss to provide the ion at *m*/*z* 353.05, which, losing a dehydrated caffeic acid, formed the citrate ion at *m*/*z* 191.01. Although these caffeic acid derivatives were not previously isolated in the *Calendula* genus, they are already known for some Asteraceae (i.e., in roots of Smallanthus sonchifolius (Poepp.) H.Rob [50] and in aerial parts of Galinsoga parviflora Cav. [51]), and displayed antiglycative activity, inhibiting the formation of AGEs (Advanced Glycation End-products) [52].

#### 3.1.2. Flavonoids

A total of 22 compounds were found to be flavonol glycosides. In particular, compounds **10**–**16** are quercetin glycosides (Figure S2). Compound **10,** with the [M−H]^−^ ion at *m*/*z* 625.1414, was putatively a quercetin-3-*O*-dihexoside. Following the neutral loss of 324 Da from the [M−H]^−^ ion, the [aglycone-H]^−^ ion at *m*/*z* 301.0346 and its abundant radical at *m*/*z* 300.0269 were provided in accordance with the glycosylation site at C-3 carbon of the flavonol. Compounds **11** and **14**, with relative [M−H]^−^ ion at *m*/*z* 595.1301 and 595.1309, were likely two isomers of quercetin-3-*O*-hexosylpentoside, giving rise to the neutral loss of 294.09 (162.05 + 132.04) Da. To confirm the presence of a pentosyl unit, a fragment ion at *m*/*z* 463.0916 (<5%) was detectable in the TOF-MS/MS spectrum of compound **14**. Quercetin-3-*O*-hexosylpentoside was recently tentatively identified in *Calendula officinalis* flowers [53]. Compounds **12** and **15**, with the [M−H]^−^ ion at *m*/*z* 609.147, were two isomers of quercetin hexosyldeoxyhexoside. Both the [M−H]^−^ ions underwent the loss of 308.11 Da to achieve the [aglycone–H]^−^ and the [aglycone–H]^•−^ ions, whereas the radical aglycone ion was favorably formed for the compound **15**. The TOF-MS/MS spectrum of this latter was superimposable with that of rutin [54]. Compound **12**, likely quercetin 3-*O*-neohesperidoside (also known as calendoflavobioside), was already identified in *C. officinalis*, along with other rhamnosyl glucosides. Indeed, calendosides I-II, isolated from *C. officinalis,* were consistent in quercetin-3-*O*-(4″-α-l-rhamnopyranosyl-β-d-glucopyranoside and quercetin-3-*O*-(3″-α-l-rhamnopyranosyl-β-d-glucopyranoside [55,56]. Compounds **13** and **16**, with [M−H]^−^ ion at *m*/*z* 463.0882 and *m*/*z* 463.0883, respectively, were quercetin-3-*O*-hexoside isomers, which underwent 162.05 Da loss in TOF-MS/MS experiment. The relative abundance of the [aglycone–H]^•–^ and [aglycone–H]^−^ was in a 3:1 ratio for compound **13**, whereas it was in a 3:2 ratio for compound **16**. The latter was likely quercetin-3-*O*-β-glucoside (isoquercitrin), previously identified in *C. arvensis* aerial parts [57], while compound **13** was hyperin [58], which was found to exert an intracellular antioxidant activity in hepatoblastoma HepG2 cell line higher than that isoquercitrin, because of the presence of specific protein receptors for galactosides [59]. Compounds **22** and **25**, with deprotonated molecular ions at *m*/*z* 505.0997 and 505.0998, respectively, were tentatively identified as acetyl derivative of quercetin-3-*O*-hexoside. Literature evidence demonstrated the isolation of derivative with 3-*O*-(2″-acetyl)-glucoside and 3-*O*-(6″-acetyl)-glucoside saccharidic moieties from *C. officinalis* flower [53]. These compounds were distinguishable based on the relative neutral losses from the [M−H]^−^ ion, which accounted to 42 Da for compound **22** and **60** Da (more favourable for the 2″-acetylderivative) for compound **25**. Quercetin was compound **35**, which displayed the [M−H]^−^ ion at *m*/*z* 301.0354, and the diagnostic TOF-MS/MS signals at *m*/*z* 273.0401 and 245.0472. The ion at *m*/*z* 178.9986 underwent CO loss to give the ion at *m*/*z* 151.0037. Further CO_2_ loss furnished the ion at *m*/*z* 107.0136.

Compounds **17**, **19**, **20**, **21**, and **24** were identified as kaempferol glycosides. Even though these compounds are poorly reported in the literature about *Calendula* spp. [60], the neutral loss of 324 Da in the TOF-MS/MS spectra of compounds **17** and **19** were in agreement with kaempferol-3-*O*-hexosyldeoxyhexoside isomers, whereas the loss of 162.05 Da suggested that compounds **20** and **21** were two hexosyl derivatives, of which kaempferol-3-*O*-glucoside was already reported in *C. officinalis* [61]. The TOF-MS/MS of compound **24**, with the base peak at *m*/*z* 285.0404, was in line with kaempferol-7-*O*-hexosyldeoxyhexoside. Isorhamnetin is the aglycone in compounds **18**, and **26**–**29**. Several isorhamnetin glycosides were previously isolated in *C. officinalis*, of which calendoflavoside and narcissin, herein identified in compounds **26** and **28**, were found abundant [54]. The TOF-MS/MS spectrum of the compound **18**, with the [M−H]^−^ ion at *m*/*z* 639.1578, was in agreement with the presence of a dihexosyl isorhamnetin. Compounds **27** and **29** were tentatively identified as isomers of isorhamentin-3*-O-*hexoside [54], whereas compounds **32** and **34** with the [M−H]^−^ ions at *m*/*z* 519.1155 and 519.1163, respectively, were tentatively identified as isorhamnetin acetylhexoside isomers. Although these metabolites were first identified in *C. arvensis*, isorhamnetin-3-*O*-(6″-acetyl-)-β-d-glucopyranoside was recently isolated among anti-acetylcholinesterase inhibitors of *C. officinalis* florets [62].

#### 3.1.3. Triterpene Saponins

A great part of the identified metabolites were triterpene saponins, already widely known as bioactive constituents of the *Calendula* genus. These compounds are known for their anti-inflammatory, antiallergic, antiulcer, immunomodulatory, cytotoxic, antimutagen, hepatoprotective, antihyperglycemic, hemolytic, antimicrobial and trypanocidal activities. It was reported that *C. officinalis* biosynthesizes oleanane saponins in all its organs, distinguishable in two series of compounds, namely the 3-*O*-monoglucoside oleanolic acid and 3-*O*-monoglucuronide oleanolic acid derivatives [63].

Recently, new bisdesmoside triterpene saponins, calendustellatosides A-E, have been described in *C. stellata*. The most representative aglycones are oleanolic, echinocystic, morolic, and mesembrianthemoidigenic acids, whereas saccharidic units are localized to C-3 and C-28 carbons [64]. At the beginning of nineties, the antipyretic and anti-inflammatory efficacy of *C. arvensis* dictated the phytochemical study of plants, resulting in the identification of these components [57], some of whom are named arvensosides [65]. Indeed, the extracts prepared from this plant were traditionally used as disinfectants, antispasmodics, diuretics, and for its diaphoretic and sedative properties [35]. To the best of our knowledge, no data about mass spectrometric behaviour of these compounds are reported and the study of their fragmentation pattern represents an excellent tool for their efficient and fast recognition in *C. arvensis*-based products. The compounds tentatively identified are reported in Figure S3.

Compound **36** was tentatively identified as 3-*O*-trihexosyl 28-*O*-echinocystic acid hexosyl ester. This metabolite was detected in the TOF-MS spectrum as formic acid adduct [M + HCOO]^−^ at *m*/*z* 1165.5691. This first evidence suggested the lack of a primarily acid site. The TOF-MS/MS spectrum highlighted the neutral loss of 162.05 Da, attributed to the (hexose-H_2_O) residue, to form the fragment ion at *m*/*z* 957.5162 by ester bond cleavage at C-28 (Figure S4). A cross ring cleavage of saccharidic moiety and the loss of 120 Da supplied the ion at *m*/*z* 837.4726, suggesting a 1–2 bond between the units [66], whereas, through the further loss of 162.05 Da, the ion at *m*/*z* 795.4604 was generated. The latter, attributed to a hexosyl-saponin, underwent H_2_O loss to achieve the ion at *m*/*z* 777.4502, and a concerted loss of (44 + 18) Da to provide the ion at *m*/*z* 633.4058, which, through further H_2_O loss, gave the ion at *m*/*z* 615.3949, while the loss of 162.05 Da generated the fragment ion at *m*/*z* 471.3505, due to the deprotonated aglycone, tentatively identified as echinocystic acid. The H_2_O and HCOOH loss from the [aglycone-H]^−^ ion favoured the ion at *m*/*z* 407.3335 formation. Based on this evidence, calendulostellaside A (3-*O*-β-d-glucopyranosyl-(1→2)-[β-d-galactopyranosyl-(1→3)]-β-d-glucopyranosyl echinocystic 28-*O*-β-D glucopyranosyl ester), previously isolated in *C. stellata* Cav., was tentatively identified. This compound was reported to be inactive (IC_50_ > 50 μM) vs. fibrosarcoma (HT1080) and human lung cancer (A549) cell lines, and able to exert a low antibacterial inhibition of *Enterococcus faecalis, Escherichia coli, Pseudomonas aeruginosa*, and *Staphylococcus epidermidis* [64].

Compound **37**, with a deprotonated molecular ion at *m*/*z* 971.4901 (C_48_H_76_O_20_), was tentatively identified as 3-*O*-(hexosyl)hexuronidyl 28-*O*-echinocystic hexosyl ester or calendasaponin B, previously isolated in flower of *C. officinalis* [67]. In fact, the [M−H]^−^ ion, following the neutral loss of 120.04 and 162.05 Da, formed the fragment ions at *m*/*z* 851.4468, and 809.4365. The latter provided an ion at *m*/*z* 747.4366 through the neutral loss of (44 + 18) Da, which could likely take place on the hexuronyl unit. Confirming this hypothesis, a further loss of 162.05 Da was detected and the ion at *m*/*z* 585.3834 was formed. The subsequent loss of 114.03 Da gave echinocystate, which lost a further 64 Da, furnishing the ion at *m*/*z* 407.3328 (Figure S5).

The TOF-MS spectrum of compound **38** displayed the [M + HCOO^−^]^−^ ion at *m*/*z* 1003.5116, while the TOF-MS/MS data were consistent with 3-*O*-dihexoxyl 28-*O*-echinocystic hexosyl ester (e.g., calendustellaside B) [64]. In fact, an abundant fragment ion at *m*/*z* 795.4572, was likely through the cleavage of the aglycone ester bond. This ion underwent the neutral loss of 162.05 Da to give the fragment ion at *m*/*z* 633.4036, which, with further water loss, generated the ion at *m*/*z* 615.3947; or, through the loss of 162.05 Da, provided the ion at *m*/*z* 471.3478, which consisted of the echinocystate (Figure S6). Compound **39**, with the deprotonated molecular ion at *m*/*z* 809.4365, was tentatively identified as 3-*O*-hexuronidyl 28-hexoxyl echinocystic acid. The TOF-MS/MS experiment emphasized the occurrence of ions deriving through 120.04 and 162.05 Da neutral loss at *m*/*z* 689.3982 and 647.3858, respectively. The hexuronidyl moiety favored the concerted loss of (44 + 18) Da to achieve the ion at *m*/*z* 585.3823, and the further loss of 114.03 Da to obtain the deprotonated aglycone ion. The ion at *m*/*z* 585.3823 could also lose 46 Da to give the ion at *m*/*z* 539.3798 (Figure S7). The compound was tentatively identified as achantopanaxoside E, previously isolated from *C. stellata* [64], and reported to exert mild inhibition of pancreatic lipase [68]. Compound **40** was tentatively identified as 3-*O*-hexuronidyl 28-*O*-mesembriantemoidigenic hexosyl ester (Figure S8). The ion [M−H]^−^ at *m*/*z* 809.4369 agreed with a constitutional isomer of a previous compound, from which it likely differs in aglycone. The loss of 162.05 Da in the TOF-MS/MS spectrum gave the ion at *m*/*z* 647.3859, and the following loss of 62 Da, favourably from glucuronate residue, generated the ion at *m*/*z* 585.3841. The latter, through the loss of 114.03 Da, provided the deprotonated aglycone at *m*/*z* 471.3508 as base peak. A decarboxylation of the ion at *m*/*z* 647.3859 gave the fragment ion at *m*/*z* 603.3941. The neutral loss of 32 Da was likely due to the loss of the hydroxymethyl functional group located in C-29 or C-30 carbons to provide the ion at *m*/*z* 439.3254 [69]. Compound **41** was putatively 3-*O*-(hydroxymethylglutarylhexosyl)hexuronidyl echinocystic acid, previously identified as the antiviral glycoside 5 [57]. The TOF-MS/MS spectrum displayed the ion at *m*/*z* 809.4382, due to the loss of hydroxymethylglutaryl moiety (−144 Da). The presence of a hexose unit in C-28 carbon was suggested by the neutral losses of 120.04 and 162.05 Da to form the ions at *m*/*z* 689.3939 and 647.3846, respectively (Figure S9). The decarboxylation and dehydration of the hexuronyl unit (−(44 + 18)Da) provided the ion at *m*/*z* 585.3841, whereas the following loss of 46 Da gave the ion at *m*/*z* 539.3767. The [aglycone-H]^−^ ion at *m*/*z* 471.3505 was through the hexuronyl moiety loss (−176.03 Da) from the ion at *m*/*z* 647.3846, and/or the loss of 114.03 Da from the ion at *m*/*z* 585.3841. The metabolite was putatively identified for the first time in the *Calendula* genus. Compound **42** was likely 3-*O*-trihexosyl 28-*O*-oleanonic acid hexosyl ester. The [M + HCOO^−^]^−^ ion at *m*/*z* 1149.5758 provided, in the TOF-MS/MS spectrum, the fragment ion at *m*/*z* 941.5228, in agreement with the ester bond cleavage and the hexosyl moiety loss (−162.05 Da) at C-28 carbon, whereas the further loss of two hexoses provided the ions at *m*/*z* 779.4672 and 617.4126. The latter ion lost H_2_O to give the ion at *m*/*z* 599.4016 (Figure S10). Compound **43** was tentatively 3-*O*-(dihexoxyl)hexuronidyl 28-*O*-oleanonic acid hexosyl ester or calenduloside H (also known as saponoside C), from the flowers of *Calendula officinalis* and aerial parts of *C. arvensis* [67]. The TOF-MS/MS spectrum displayed the deprotonated molecular ion at *m*/*z* 955.5031, from which the ion at *m*/*z* 793.4472, through hexosyl moiety loss, was formed, and further underwent 62 Da loss to provide the ion at *m*/*z* 731.4451, or hexosyl moiety loss, to obtain the weak ion at *m*/*z* 631.3906. Analogously, the loss of 162.05 Da from the ion at *m*/*z* 731.4451 formed that at *m*/*z* 569.3895, which could provide the ions at *m*/*z* 551.3783 and 455.3557, by H_2_O and 96 Da losses, respectively (Figure S11). The compound **44** was tentatively 3-*O*-dihexosyl 28-*O*-oleanonic acid hexosyl ester, likely arvensoside A [70]. The [M + HCOO^−^]^−^ at *m*/*z* 987.5207 lost 162.05 Da to give the ion at *m*/*z* 779.4669, which, for further loss of 162 Da, gave the ion at *m*/*z* 617.4121. Further H_2_O loss gave the ion at *m*/*z* 599.4011, whereas the [aglycone-H]^−^ ion was formed as base peak after the loss of 162.05 Da (Figure S12).

The [M + HCOO^−^]^−^ for the compound **45** at *m*/*z* 825.4661 was in accordance with the 3-*O*-hexosyl 28-*O*-oleanonic acid hexosyl ester. The TOF-MS/MS spectrum displayed the product ion at *m*/*z* 617.4099 (formed by the loss of 162.05 Da from the undetectable deprotonated molecular ion, with a theoretical *m*/*z* at 779.4587), which gave the fragment ion at *m*/*z* 455.3564 for further loss of 162.05 Da. The loss of 180 Da from the [M−H]^−^ putative ion provided the base peak at *m*/*z* 599.3986. Even though the metabolite shares its molecular formula with arvensoside B, the absence of information about the deprotonated molecular ion allowed us to hypothesize a constitutional isomer of the compound previously isolated, glycosylated at C-3 and C-28 carbons (Figure S13). Accordingly, silphioside B (3-*O*-β-d-glucopyranosyl oleanolic acid 28-*O*-β-d-glucopyranosyl ester) was isolated from *C. stellata* [64]. Compound **46** was tentatively identified as 3-*O*-hexuronidyl oleanonic acid hexosyl ester. The TOF-MS/MS spectrum displayed the ion at *m*/*z* 631.3922, formed for loss of hexose unit (−162.05 Da) from the deprotonated molecular ion. The decarboxylation and dehydration of the hexuronyl unit (44 + 18)Da formed the fragment ion at *m*/*z* 569.3895, which could lose 72 Da to provide the ion at *m*/*z* 497.3677, or 114.03 Da to form the ion at *m*/*z* 455.3562. The latter could be also obtained by the neutral loss of 176.03 Da from the ion at *m*/*z* 631.3922 (Figure S14). The compound is likely calenduloside F, previously isolated from the root of *C. officinalis* [71]. Compound **47**, with the deprotonated molecular ion at *m*/*z* 835.4525, was tentatively identified as 3-*O-h*exuronidyl 28-O-oleanonic acid acetylhexosyl ester. The TOF-MS/MS spectrum displayed the product ion at *m*/*z* 793.4436, generated from the loss of an acetylhexose-H_2_O (−204.06 Da) unit. The fragment ion at *m*/*z* 569.3898 was by decarboxylation and dehydration of hexuronyl moiety (44 + 18) Da. The subsequential loss of 72 Da was observed for cross-ring cleavage to provide the fragment ion at *m*/*z* 497.3677, which in turn generated, by loss of 42 Da, the fragment ion at *m*/*z* 455.3556 (Figure S15). This metabolite was identified for the first time in *Calendula* genus. Compound **48** was tentatively identified as 3-*O*-(hydroxymethylglutaryl)hexuronidyl 28-*O*-oleanonic acid hexosyl ester. Its TOF-MS/MS spectrum showed the ion at *m*/*z* 793.4457, generated through the loss of the hydroxymethylglutaryl unit (−144 Da) from the deprotonated molecular ion, and the ion at *m*/*z* 631.3909, due to the further 162.05 Da neutral loss. The fragment ion at *m*/*z* 569.3899 was provided by decarboxylation and dehydration of the hexuronyl unit. Finally, in the TOF-MS/MS spectrum, the deprotonated aglycone moiety at *m*/*z* 455.3559, putatively identified as oleanolic acid, was detectable, together with the ion at *m*/*z* 437.3425, due to further water loss (Figure S16). The compound was previously isolated in *Calendula arvensis* as arvensoside C. The compound **49** with the deprotonated molecular ion at *m*/*z* 791.4258 was putatively identified as 3-*O*-(hydroxymethylglutaryl)hexuronidyl echynocistic acid. The TOF-MS/MS spectrum highlighted the fragment ion at *m*/*z* 647.3850, formed by the loss of the hydroxymethylglutaryl unit (−144 Da). The presence of a glucuronate moiety was suggested by the further neutral loss of 176.03 Da, which provided the ion at *m*/*z* 471.3516 and *m*/*z* 407.3341 (Figure S17).

#### 3.1.4. Guidelines for the Straightforward Identification of Triterpene Saponins by HR-MS/MS Tools

The TOF-MS and TOF-MS/MS data allow us to describe some guidelines that favour the rapid identification of this class of compounds in complex mixtures. In fact, it is observed that when the compound is glycosylated, both in the alcoholic function in C-3 and in the carboxylic function in C-28, only the adduct with formic acid is detectable in the TOF-MS spectrum, and, more likely, the first fragment ion observed in the TOF-MS/MS spectra is the result of the loss of a dehydrated hexose sugar from the undetected molecular deprotonated ion. The latter was well distinguishable in the TOF-MS spectra of compounds in which an oxidized sugar occurs, or in compounds whose C-28 carbon was not esterified. Compounds with hexuronate, beyond neutral losses of 162.05 Da, attributable to [hexose-H_2_O] residues, displayed the characteristic loss of 176.03 Da. Hexuronic acid was also recognized based on the concerted loss of 62 Da, which was through decarboxylation (−44 Da) and dehydration (−18 Da), and the loss of a [hexuronic acid-(2H_2_O + CO_2_)] moiety. Once an acyl moiety was present (e.g., hydroxymethylglutarate) and linked on a sugar part, it was quickly lost from the deprotonated molecular ion.

#### 3.1.5. Other Compounds

Compound **1** was likely malic acid, and compound **2** was quinic acid. In particular, the molecular deprotonated ion at *m*/*z* 133.0140 for compound **1**, gave, following the loss of water, the ion at *m*/*z* 115.0036 as base peak. The TOF-MS spectrum of compound **3** displayed the deprotonated molecular ion at *m*/*z* 341.1108 (C_12_H_22_O_11_). The compound was tentatively identified as a dihexose. In the TOF-MS/MS spectrum, the cleavage of *O*-glycosidic bond and the loss of a hexose unit (−162.05 Da) generated the fragment ion at *m*/*z* 179.0562. Confirming its saccharidic nature, it was detected in the fragment ions at *m*/*z* 119.0348 and at *m*/*z* 89.0244, which were formed by the cross-ring cleavage of the saccharidic unit and the corresponding neutral loss of 60 Da and 90 Da. This compound was tentatively identified as trehalose, a disaccharide with a glycosidic bond α(1→1′), already reported in the literature as abundant in *C. officinalis* [72]. The non-reducing nature of saccharide agreed to the presence of the fragment ion at *m*/*z* 179.0562. In fact, non-reducing sugars are distinguished from those reducing (e.g., lactose and maltose) in tandem mass spectrometry by the absence of fragments with a higher *m*/*z* ratio, and the fragmentation involves the acetalic bond directly [73]. Trehalose acts, in many organisms, as a source of energy or as a protective agent against the effects of freezing or dehydration. Its physical and/or chemical features allow it to differ from other sugars, and to be favourably engaged as an ingredient for many food, healthcare and, pharmaceutical products [74]. Studies in *C. officinalis* seedlings observed that the exposition at low temperature strongly increases the content of this osmoprotectant [72]. The TOF-MS spectrum of compound **4**, displaying the deprotonated molecular ion at *m*/*z* 153.0195, was in accordance with the dihydroxybenzoic acid. In fact, the decarboxylation of the deprotonated molecular ion generated the fragment ion at *m*/*z* 109.0287 and the radical ion at *m*/*z* 108.0214, attributable to the presence of a diphenolic moiety. In this context, protocatechuic acid or 3,4-dihydroxybenzoic acid was previously identified in the methanolic extract of *C. officinalis* flowers [75]. Compound **5**, with the deprotonated molecular ion at *m*/*z* 285.0619, was the pentosyl derivative of the previous one. In particular, the loss of 132 Da generated, in the TOF-MS/MS spectrum, the fragment ion at *m*/*z* 153.0190 and the radical ion at *m*/*z* 152.0111. The decarboxylation of the radical ion provided the abundant formation of the ion at *m*/*z* 108.0214. This compound was previously identified in a methanolic extract of *C. arvensis* aerial parts [39]. The presence of hydroxybenzoic acid and its derivatives, such as protocatechuic acid hexoside and a syringic acid derivative, was also observed in the subspecies *lusitanica* (Boiss.) Ohle and *algarbiensis* (Boiss.) Nyman of *C. suffruticosa*. [39].

### 3.2. Multivariate Analysis

**Figure 3:**
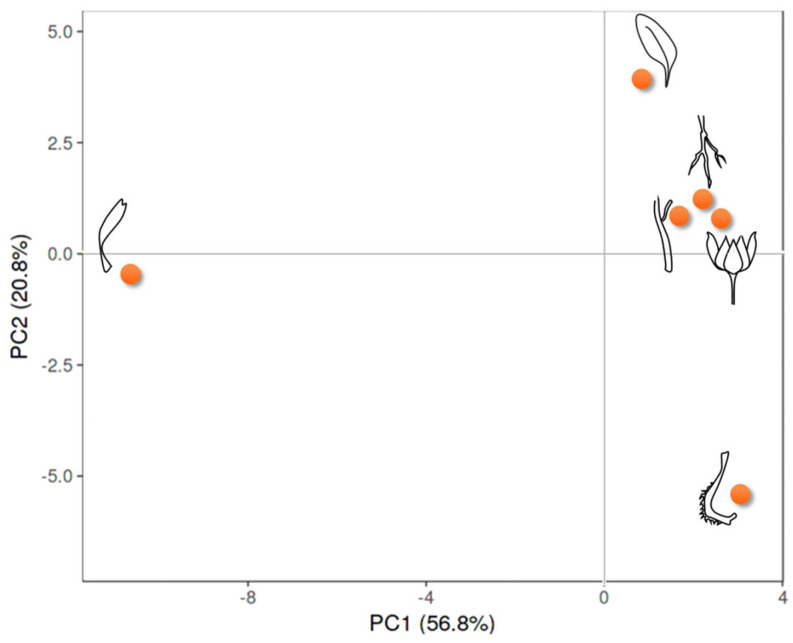
PCA of tentatively identified metabolites in six organs of *Calendula arvensis*.

A multivariate analysis approach was adopted to explore and clarify the quali-quantitative compositive data analysis of each organ. In particular, the principal component analysis (PCA), considering the two principal components (PC) that described 77.6% of the total variance with PC1 and PC2, representing 20.8% and 56.8%, respectively, highlighted the compositive distinctiveness of florets positioned at the end of the negative score of the PC1 axis. On the contrary, all the other organs grouped in the positive score, even if fruits were at the end of the negative score of PC2 and leaves in the positive one; the root extract was positively correlated with those of stems and bracts (Figure 3).

**Figure 4:**
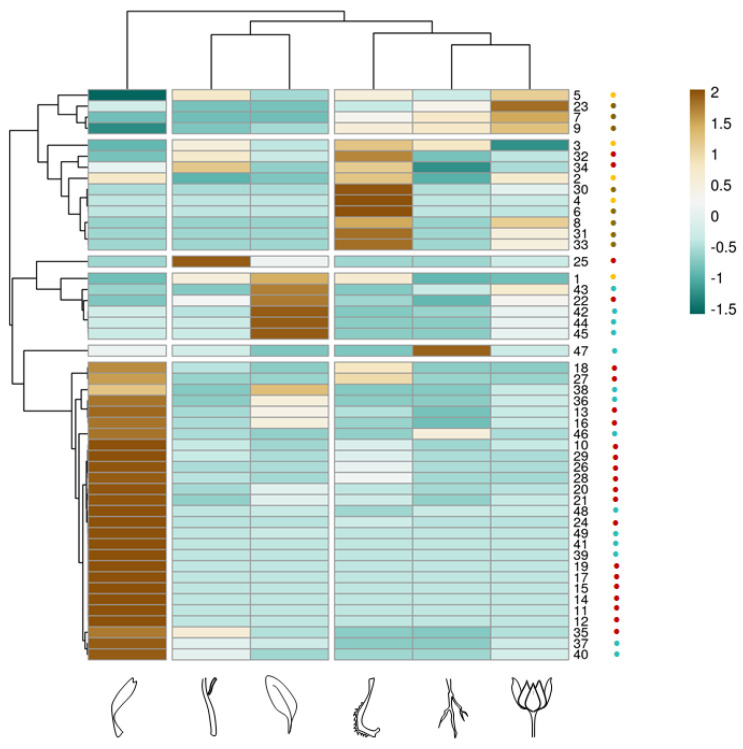
Heatmap of the compounds in the alcoholic extracts from the different organs of Calendula arven-sis: • hydroxycinnamoyl compounds; • flavonoids; • triterpene saponins; • other compounds. Clustering of the investigated samples is displayed above the heatmap. In the ClustVis hierar-chical clustering tool, rows and columns are clustered by means of correlation distance and aver-age linkage.

The heatmap of the alcoholic extract of *C. arvensis* organs clearly evidenced the cluster segregations of the different quali-quantitative compound compositions in relation to each analysed organ (Figure 4), confirming for the latter the distinctiveness of florets obtained, based on the PCA. Moreover, the other subcluster of organs matched to leaves and stems on the one hand, and roots and bracts on the other. The basis that drives the separation is to achieve homogeneous elements. Considering each individual class of the compounds, it appears clear that fruits and bracts are distinguishable for their higher content of hydroxycinnamic derivative. Florets are rich in flavonoid glycosides and triterpenic saponins. The latter are completely absent in fruits, which are characterized for the greater presence of flavonol acetylglycosides.

### 3.3. Antioxidant Activity of Calendula arvensis Alcoholic Extracts

Data from the in vitro antiradical capability of the alcoholic extracts of the *C. arvensis* organs were preliminarily analysed by cluster analysis, to explore the degree of dissimilarity values between test types and plant organs. An average linkage agglomeration criterion and Jaccard Index as dissimilarity coefficient were applied to each (6 organs × 5 concentrations) of the three data matrix of radical scavenging activity (ABTS, PFRAP, and DPPH).

**Figure 5:**
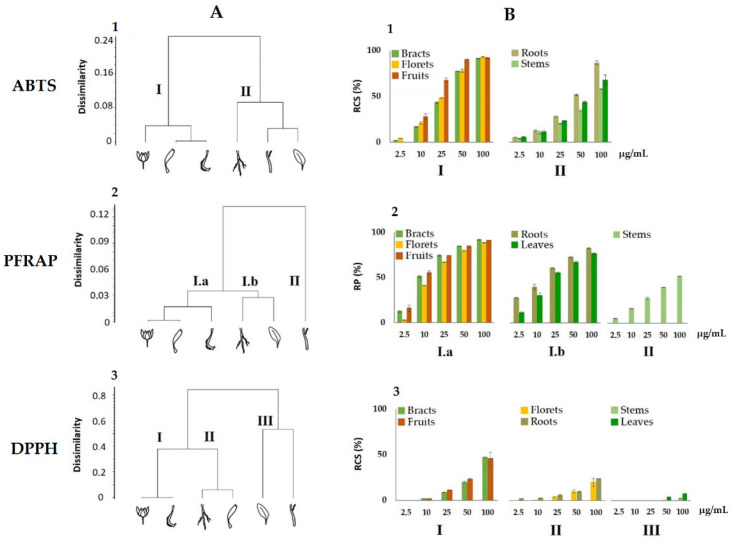
Dendrograms (**A**) and activity (**B**) from antioxidant assays (ABTS, PFRAP, and DPPH) carried out on the alcoholic extracts of the *Calendula arvensis* organs. RSC, Radical Scavenging Capacity; RP, Reducing Power. Data are expressed as the mean ± SD of two experiments, independently carried out, each of which in triplicate.

The obtained dendrograms (Figure 5A) clearly demonstrated different clustering patterns of the organs’ alcoholic extract, in relation to the antioxidant test used. The dendrogram obtained for the ABTS data assay highlighted two clusters (Figure 5A1), which have a dissimilarity value of 24% among them, with the first including bracts, florets, and fruits, while the second grouping was root, stem, and leaf extracts. On the contrary, the cluster analysis for PFRAP assay displayed two main clusters (Figure 5A2), with the first one characterized in turn by two subclusters including bracts, florets, and fruits on one side, and roots and leaves on the other (Figure 5A2 (I.a)), while the second consisted only of stems.

The dendrogram, relating to the results of the DPPH assays, displayed three clusters (Figure 5A3): the first group included bracts and fruit extracts, the second, comprised of roots and flowers, and the third cluster consisted of stem and leaf extracts.

In a second step, the antioxidant activity data were organized and depicted according to the obtained clusters of each dendrogram, respectively (Figure 5B). It can be observed in the ABTS and PFRAP tests that the activity values of bracts, florets, and fruits are grouped in a single cluster, I and Ia respectively, which resulted in the most active and with a similar pattern in relation to concentrations. Stems, leaves, and roots are instead clustered together both in ABTS and PFRAP, cluster II and Ib respectively, except for the PFRAP, where the stems are included in a separate group, displaying the lowest activity values. A different behaviour was highlighted for the DDPH test, where the lowest values were measured with a progressive decrease in activity from cluster I (bracts and fruits) to the lowest of the III (stems and leaves). The analysed data reveals that the classes of metabolites in the alcoholic extracts, responsible for the observed antioxidant activity, lead to a different response depending on assay used, but mainly on the quali- and quantitative composition of the extracts. In particular, the highest activity of bracts and fruits can be in agreement with the large amount of hydroxycinnamoyl compounds, such as chlorogenic acid and dicaffeoyl quinic acid for bracts, and tricaffeoyl citric acid in fruit extract. In fact, these compounds contain catechol moiety in their structure, which is highly reactive, based on its two exchangeable hydrogen atoms. They exhibit an antioxidant activity more than that of glycosylated flavonoids, particularly abundant in the florets extract, since the glycosylation reduces their antioxidant activity when compared to that of their respective aglycones [49].

The interest in the *Calendula* genus has always been high but, from a scientific point of view, if the chemistry and bioactivity of *C. officinalis*, also elected the herb of the year in 2008 by the International Herb Association [76], have been widely investigated, little attention has been given, if not in local research, to *C. arvensis*. The latter, which could familiarly be referred to as the ugly half-sister of the pot marigold, shares its phytochemical goodness. Although *C. arvensis* flower and leaf extracts were extensively studied for their antioxidant efficacy [77], our study highlighted that the biological activity commonly ascribed to “flowers” is largely attributable also to a specific part of the inflorescence, including involucral bracts and fruits. Thus, the careful examination of the bioactivity of the different plant parts is also necessary to increase its potential for use [78].

**Figure 6:**
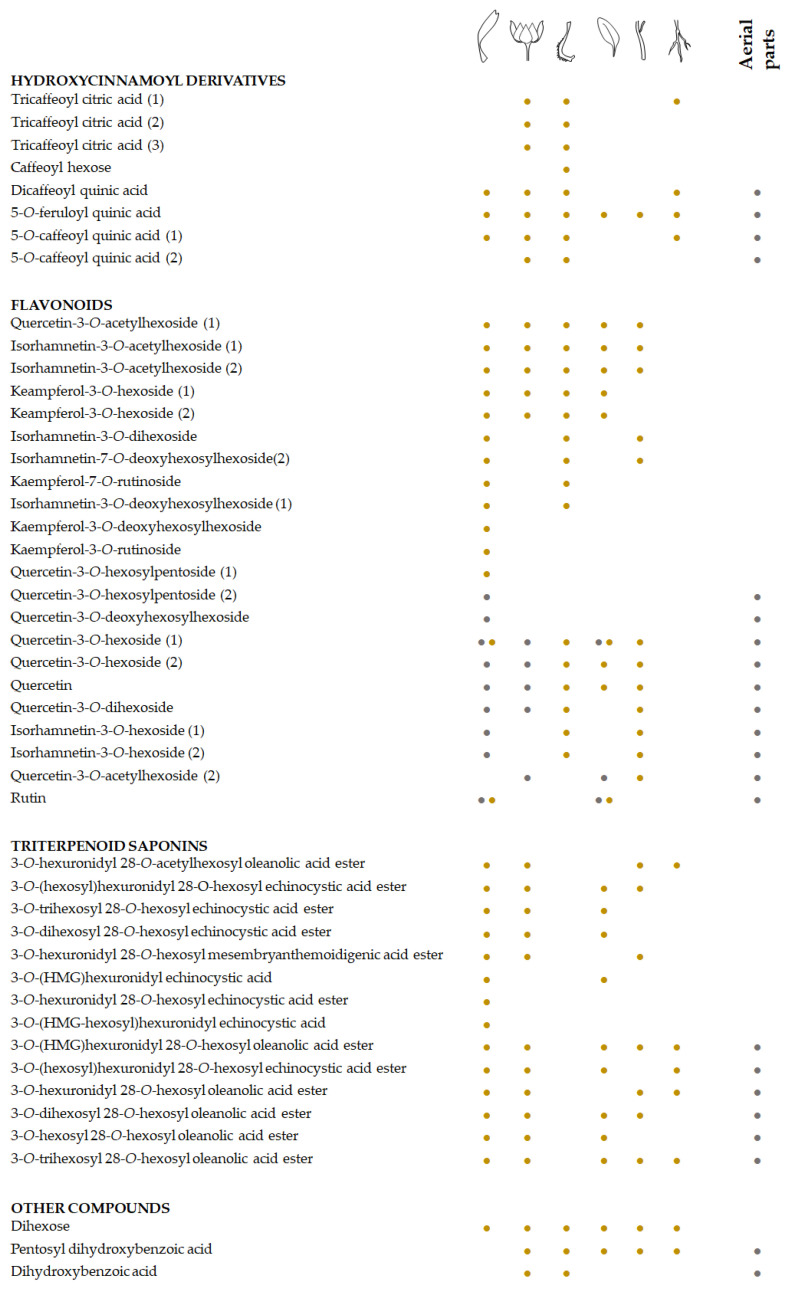
Comparison of the specialized metabolites herein identified in the different organs from Calendula arvensis (•) and in previous literature reports (•) [38,39,57,65,77].

The diversity in flavonol glycosides has not been highlighted before in the few studies conducted, relating to the phytochemical aspects of the species (Figure 6). In this context, an in-depth study of the current literature highlights that a large portion of the compounds identified have not been reported before as constituents of *C. arvensis*, where several studies underline their presence in other species of the same genus, even if different from the more attentive *C. officinalis*. However, recently, some beneficial properties of the *C. arvensis* are enhancing the need to thoroughly detail its chemical constitution, also with the objective to fully exploit its properties on human and animal health. The hypoglycaemic activity, exerted mainly through enzyme inhibition, of the aqueous and methanolic extracts of *C. arvensis* flowers have been demonstrated [79]. The inhibitory activity against α-amylase, α-glucosidase, and β-galactosidase was ascribed to caffeic acid and its derivatives. Indeed, considering compositive information herein acquired, these compounds are mainly abundant in the fruits of the species. The fruit organ lacks triterpene saponins. The latter were broadly studied for their gastroprotective, antiviral, antimutagenic, and anti-inflammatory activities, and their recovery in bracts, as well as in leaves and stems, making these organs an exploitable source. In light of this, and taking into account that *C. arvensis*, from an ecological point of view, displays a wide diffusion strategy even outside its original range [23], it is interesting to evaluate that the availability to recover its bioactive compounds could be a tool also to counteract the high weed risk assessment for the species [80]. In this context, and considering process and product sustainability, the chemical compositional study of *C. arvensis* points to new scenarios in which the use of the plant for food and/or nutraceutical purposes is also fully configured. The data acquired provide a valuable tool for revalorizing this wild food species, preserving its traditional uses, and improving the Mediterranean diet assortment.

## 4. Conclusions

Wild edible plants are a great source of bioactive specialized metabolites whose intake could be beneficial for humans, but the knowledge on these plants, broadly consumed as part of local dishes, is still not enough. Wild plants, whose leaves, flowers, or fruits are edible, can be particularly tasty. This is mainly true for the native, pleasant looking species *C. arvensis* (Vaill.) L., which represents a strong competitor in agricultural, anthropogenic, and natural systems. The wide range of distribution and locally high coverage of this species is due to its ability to adapt to different environments, also thanks to its richness in specialized metabolites. Furthermore, considering its local use in the food and cosmetic sectors, the valuable use of all its organs represents a feasible strategy. Herein, the UHPLC-Q*q*TOF-MS/MS analysis of the alcoholic extracts from the species organs, properly dissected, has particularly emphasized that the knowledge of the phytochemistry of this species is far from being fully known. On the other hand, since the phytochemical diversity of the various organs is similar to other species of the genus *Calendula*, which have a more marked economic impact, *C. arvensis* has a potential for use that is not fully exploited. The data acquired highlighted that each organ is a reservoir of specific classes of substances. Not disregarding the botanical role that each organ plays, and investigating finely its composition, fruits, and bracts of *C. arvensis*, which share a great part of hydroxycinnamoyl compounds, are highlighted as a fascinating source for further exploration within and beyond the food field. The research data are a stimulus for further investigation aimed at highlighting, on the one hand, the phytochemical-environmental aspects of this species, also considering its harvesting in different areas, and on the other hand, to deepen the health aspects of its various organs in order to consider the use of *C. arvensis* not only for its florets, which add a touch of color and fragrant aromas to salads or other dishes, or even for its leaves, which could be boiled or not, to vary and/or characterize the flavor of local dishes. Therefore, the data acquired are nothing more than a starting point, and, based on the chemical diversity of the polar constituents, a systematic organ-specific investigation of the apolar component will be pursued.

## Figures and Tables

**Table 1:** Metabolites tentatively identified in the alcoholic extracts from the different organs of *Calendula arvensis*. RT, Retention Time; RDB, Ring Double Bond equivalent value.

Peak n°	t_R_ (min)	Formula	RDB	[M−H]^−^ (*m*/*z*) Found	[M−H]^−^ (*m*/*z*) calcd.	ppm	MS/MS (*m*/*z*)	Tentative Assignment
**1**	0.311	C_4_H_6_O_5_	2.0	133.0140	133.0142	−1.9	133.0139, 115.0036 (100)	Malic acid
**2**	0.317	C_7_H_12_O_6_	2.0	191.0557	191.0561	−2.2	191.0556 (100), 111.0083, 93.0344, 85.0294	Quinic acid
**3**	0.322	C_12_H_22_O_11_	2.0	341.1108 377.0860 [M + Cl^−^]^−^	341.1089	−2.2	341.1077, 179.0562, 119.0348, 113.0247, 89.0247	Dihexose
**4**	1.174	C_7_H_6_O_4_	5.0	153.0195	153.0193	1.1	109.0287, 108.0335	Dihydroxybenzoic acid
**5**	1.824	C_12_H_14_O_8_	6.0	285.0619	285.0616	1.1	285.0605, 153.0190, 152.0111, 108.0214	Pentosyl dihydroxybenzoic acid
**6**	2.080	C_15_H_18_O_9_	7.0	341.0880	341.0878	0.6	251.0570, 179.0354, 161.0252 (100), 135.0456	Caffeoyl hexose
**7**	3.799	C_16_H_18_O_9_	8.0	353.0886 707.1848	353.0878	2.2	191.0559 (100)	5*-O-*Caffeoylquinic acid (1)
**8**	4.627	C_16_H_18_O_9_	8.0	353.0877	353.0878	−0.3	191.0561 (100)	5-*O-*Caffeoylquinic acid (2)
**9**	6.582	C_17_H_20_O_9_	8.0	367.1038	367.1035	0.9	193.0513, 191.0560 (100), 173.0453	5-*O-*Feruloyl quinic acid
**10**	8.179	C_27_H_30_O_17_	13.0	625.1414	625.1410	0.6	625.1422 (100), 301.0346, 300.0269, 271.0237, 255.0294	Quercetin-3-*O*-dihexoside
**11**	8.771	C_26_H_28_O_16_	13	595.1301	595.1305	−0.6	595.1318, 301.0349, 300.0253, 271.0234, 255.0291	Quercetin-3*-O*-hexosylpentoside
**12**	9.116	C_27_H_30_O_16_	13.0	609.1472	609.1461	1.8	609.1477, 301.0348, 300.0270 (100), 271.0235, 255.0292	Quercetin-3-*O*-hexosyldeoxyhexoside
**13**	9.386	C_21_H_20_O_12_	12.0	463.0882	463.0882	0	463.0905, 301.0358 300.0281 (100), 271.0253, 255.0300	Quercetin-3*-O*-hexoside (1)
**14**	9.326	C_26_H_28_O_16_	13.0	595.1309	595.1305	0.7	595.1303, 463.0916 (<5%), 301.0340, 300.0260 (100), 271.0226, 255.0306	Quercetin-3*-O*-hexosylpentoside
**15**	9.473	C_27_H_30_O_16_	13.0	609.1473	609.1461	1.8	609.1484, 301.0253 300.0275 (100), 271.0246, 255.0295	Rutin
**16**	9.692	C_21_H_20_O_12_	12.0	463.0883	463.0882	0.2	463.0879, 301.0341 300.0267 (100), 271.0238, 255. 0285	Quercetin-3-*O*-hexoside (2)
**17**	10.024	C_27_H_30_O_15_	13.0	593.1507	593.1512	−0.8	593.1536 (100), 285.0395, 284.0314, 255.0287	Kaempferol-3*-O-*hexosyldeoxyhexoside
**18**	10.024	C_28_H_32_O_17_	13.0	639.1578	639.1567	1.8	639.1594, 315.0507 (100), 300.0267, 314.0426, 299.0187, 271.0237, 255.0287	Isorhamnetin-3*-O-*dihexoside
**19**	10.885	C_27_H_30_O_15_	13.0	593.1527	593.1512	2.5	593.1444, 285.0403, 284.0323 (100), 255.0298, 227.0335	Kaempferol-3*-O-*rutinoside
**20**	10.338	C_21_H_20_O_11_	12.0	447.0927	447.0933	−1.3	447.0925, 285.0388, 284.0316 (100), 255.0283, 227.0333	Keampferol-3-*O*-hexoside (1)
**21**	10.974	C_21_H_20_O_11_	12.0	447.0930	447.0933	−0.6	447.0915, 285.0391, 284.0314 (100), 255.0280, 227.0334	Keampferol-3-*O*-hexoside (2)
**22**	10.424	C_23_H_22_O_13_	13.0	505.0997	505.0988	1.9	505.1005, 463.0879, 301.0351, 300. 0273 (100), 271.0253, 255.0287; 243.0315; 151.0027	Quercetin-3-*O*-acetylhexoside (1)
**23**	10.615	C_25_H_24_O_12_	14.0	515.1197	515.1195	0.4	353.0878, 191.0561 (100), 179.0346, 135.0450	Dicaffeoyl quinic acid
**24**	10.872	C_27_H_30_O_15_	13.0	593.1515	593.1512	0.5	593.1436, 285.0404 (100), 284.0324, 255.0290	Kaempferol-7*-O-*rutinoside
**25**	10.920	C_23_H_22_O_13_	13.0	505.0998	505.0988	2.0	505.1008, 445.0825, 301.0345, 300.0271, 271.0264, 255.0293, 174.9562	Quercetin-3-*O*-acetylhexoside (2)
**26**	11.013	C_28_H_32_O_16_	13.0	623.1634	623.1618	2.6	623.1645, 315.0512, 314.0437 (100), 300.0275, 299.0199, 243.0300	Isorhamnetin-3*-O-*hexosyldeoxyhexoside (1)
**27**	11.247	C_22_H_22_O_12_	12.0	477.1043	477.1029	0.9	477.1037, 315.0497, 314.0422 (100), 300.0258, 299.0181, 285.0386, 271.0234, 243.0287, 242.0207	Isorhamnetin-3-*O*-hexoside (1)
**28**	11.366	C_28_H_32_O_16_	13.0	623.1629	623.1618	1.8	623.1656, 315.0514 (100), 314.0436, 299.0200, 271.0253, 255.0297, 243.0298	Isorhamnetin*-*3*-O-*hexosyldeoxyhexoside (2)
**29**	11.442	C_22_H_22_O_12_	12.0	477.1043	477.1029	0.9	477.1053, 315.0501, 314.0430 (100), 299.0193, 285.0396, 271.0244, 243.0291, 242.0217	Isorhamnetin-3-*O*-hexoside (2)
**30**	11.617	C_33_H_28_O_17_	20	695.1270	695.1254	2.3	695.1256, 533.0958, 371.0620, 209.0299 (100), 191.0186	Tricaffeoyl citric acid (1)
**31**	11.833	C_33_H_28_O_17_	20	695.1267	695.1254	1.9	695.1305, 533.0978, 371.0633, 353.0513, 209.0303 (100), 101.0191, 85.0293	Tricaffeoyl citric acid (2)
**32**	11.963	C_24_H_24_O_13_	13.0	519.1155	519.1148	2.1	519.1174, 315.0507, 314.0432 (100), 300.0279, 299.0192, 285.0397, 271.0241	Isorhamnetin-3-O-acetylhexoxide
**33**	12.026	C_33_H_28_O_17_	20	695.1268	695.1254	2.1	695.1284, 533.0977, 371.0616, 353.0487, 209.0297 (100), 191.0298	Tricaffeoyl citric acid (3)
**34**	12.164	C_24_H_24_O_13_	13.0	519.1163	519.1148	3.6	519.1161, 315.0499, 314.0423 (100), 300.0264, 299.0192, 285.0394, 271.0237	Isorhamnetin-3-*O*-acetylhexoxide
**35**	13.538	C_15_H_10_O_7_	11.0	301.0354	301.0354	0.1	301.0349, 273.0401, 245.0472, 178.9986, 151.0037 (100), 107.0136	Quercetin
**36**	15.960	C_54_H_88_O_24_	11.0	[M + HCOO^−^]^−^ 1165.5691 [M + Cl^−^]^−^ 1155.5410	1119.5593	n.c.	957.5162, 837.4726, 795.4604 (100), 777.4502, 733.4599, 633.4058, 615.3949, 505.3716, 471.3505, 407.3335, 161.0449, 119.0346, 113.0243	3-*O*-trihexosyl 28-*O*-echinocystic acid hexosyl ester
**37**	16.229	C_48_H_76_O_20_	11.0	971.4901	971.4857	4.5	971.4929, 851.4468, 809.4365 (100), 747.4366, 647.3833, 585.3834, 513.3617, 471.3478, 409.3477, 407.3328, 157.0152, 119.0344, 113.0240	3-*O*-(hexosyl)hexuronidyl 28-*O*-echinocystic acid hexosyl ester
**38**	16.603	C_48_H_78_O_19_	10.0	[M + HCOO^−^]^−^ 1003.5116	957.5065	n.c.	795.4572 (100), 733.4632, 633.4036, 615.3947, 471.3478, 161.0433, 101.0245	3-*O*-dihexosyl 28-*O*-echinocystic acid hexosyl ester
**39**	16.758	C_42_H_66_O_15_	10.0	809.4365	809.4388	4.5	809.4373, 689.3982, 647.3858, 585.3823, 539.3798, 471.3512, 425.3427, 407.3340 (100), 391.3033, 245.1536, 113.0244	3-*O*-hexuronidyl 28-*O*-hexoxyl echinocystic acid
**40**	17.088	C_42_H_66_O_15_	10.0	809.4369	809.4388	3.8	809.4419, 647.3859, 603.3941, 485.3646, 471.3508 (100), 469.3355, 453.3399, 439.3254, 393.3168, 113.0243	3-*O-*hexuronidyl 28-*O*-mesembryanthemoidigenic hexosyl ester
**41**	17.939	C_48_H_74_O_45_	12.0	953.4785	953.4752	3.6	809.4382, 689.3939, 647.3846 (100), 585.3841, 539.3767, 471.3505, 407.3328, 409.3488, 391.3006, 113.0242	3-*O*-(hydroxymethylglutarylhexosyl)hexuronidyl echinocystic acid
**42**	18.377	C_54_H_88_O_23_	11.0	[M + HCOO^−^]^−^ 1149.5758	1103.5644	1.9	941.5228, 779.4672, 617.4126, 599.4016, 551.3785, 455.3568 (100)	3-*O*-trihexosyl 28-*O*-oleanonic acid hexosyl ester
**43**	18.900	C_48_H_76_O_19_	11.0	955.4946	955.4908	4.0	955.5031, 793.4472, 731.4451, 631.3906, 571.3697, 569.3895, 551.3790 (100), 497.3666, 483.3509, 455.3565, 453.3405, 437.3444, 407.3332	3-*O*-(hexosyl)hexuronidyl 28-*O*-oleanonic acid hexosyl ester
**44**	19.211	C_48_H_78_O_18_	10.0	[M + HCOO^−^]^−^ 987.5207	941.5115	n.c.	779.4669, 617.4121 (100), 599.4011, 455.3558	3-*O*-dihexosyl 28-*O*-oleanonic acid hexosyl ester
**45**	19.273	C_42_H_68_O_13_	9.0	[M + HCOO^−^]^−^ 825.4661	779.4587	n.c	617.4100, 599.4001 (100), 455.3538	3-*O*-hexosyl 28-*O*-oleanonic acid hexosyl ester
**46**	19.778	C_42_H_66_O_14_	10.0	793.4417	793.4418	4.7	793.4419, 631.3922, 569.3895, 497.3670, 455.3562 (100), 437.3439	3-*O*-hexuronidyl 28-*O*-oleanonic acid hexosyl ester
**47**	20.713	C_44_H_68_O_15_	11.0	835.4520	835.4485	4.1	793.4436, 631.3912, 569.3898 (100), 551.3805, 497.3677, 455.3556, 437.3447	3-*O-*hexuronidyl 28-*O*-oleanonic acid acetylhexosyl ester
**48**	20.888	C_48_H_74_O_18_	12.0	937.4846	937.4802	4.7	793.4457, 673.4011, 631.3909, 569.3899 (100), 551.3770, 497.3672, 455.3559, 437.3435	3-*O*-(hydroxymethylglutaryl)hexuronidyl 28-*O*-oleanonic acid hexosyl ester
**49**	21.821	C_42_H_64_O_14_	11.0	791.4258	791.4223	4.4	647.3850, 571.3670, 471.3516, 469.3603, 407.3341 (100), 391.3022, 116.0116, 113.0239	3-*O*-(hydroxymethylglutaryl)hexuronidyl echynocistic acid

## Data Availability

The data are included in this manuscript and in its Supplementary Materials.

## Supplementary Materials

Supporting information can be downloaded at: https://www.mdpi.com/article/10.3390/foods11030247/s1. Figure S1: (a) TOF-MS/MS spectrum of the compounds (i) **30**, (ii) **31**, and (iii) **33**; (b) tentative fragmentation pathway of the theoretical [M−H]^−^ ion at *m*/*z* 695.1254; Figure S2: TOF-MS/MS spectrum of the quercetin glycosides from the florets alcoholic extract (a) **10**, (b) **11**, (c) **12**, (d) **13**, (e) **14**, (f) **15**, (g) **16**, and (h) **22**. Neutral losses of 324.10 (**10** and **12**), 294.09 (**11** and **14**), 308.11 (**12** and **15**), 162.05 (**13** and **16**), and 204.06 (**22**) allowed the glyconic moiety to be recognized; Figure S3: Chemical structures of triterpene saponins (**36**–**49**) tentatively identified in the *Calendula arvensis* alcoholic extracts. Hex, hexose; Hexu, hexuronic acid; Achex, acetylhexose; HMG, Hydroxymethylglutaric acid; Figure S4: (a) TOF-MS/MS spectrum and (b) tentative fragmentation pathway of the theoretical [M−H]^−^ ion for compound **36**. Figure S5: (a) TOF-MS/MS spectrum and (b) tentative fragmentation pathway of the theoretical [M−H]^−^ ion for compound **37**. Figure S6: (a) TOF-MS/MS spectrum and (b) tentative fragmentation pathway of the theoretical [M−H]^−^ ion for compound **38**. Figure S7: (a) TOF-MS/MS spectrum and (b) tentative fragmentation pathway of the theoretical [M−H]^−^ ion for compound **39**. Figure S8: (a) TOF-MS/MS spectrum and (b) tentative fragmentation pathway of the theoretical [M−H]^−^ ion for compound **40**. Figure S9: (a) TOF-MS/MS spectrum and (b) tentative fragmentation pathway of the theoretical [M−H]^−^ ion for compound **41**. Figure S10: (a) TOF-MS/MS spectrum and (b) tentative fragmentation pathway of the theoretical [M−H]^−^ ion for compound **42**. Figure S11: (a) TOF-MS/MS spectrum and (b) tentative fragmentation pathway of the theoretical [M−H]^−^ ion for compound **43**. Figure S12: (a) TOF-MS/MS spectrum and (b) tentative fragmentation pathway of the theoretical [M + HCOO^−^]^−^ ion for compound **44**. Figure S13: (a) TOF-MS/MS spectrum and (b) tentative fragmentation pathway of the [M + HCOO^−^]^−^ ion for compound **45**. Figure S14: (a) TOF-MS/MS spectrum and (b) tentative fragmentation pathway of the theoretical [M−H]^−^ ion for compound **46**. Figure S15: (a) TOF-MS/MS spectrum and (b) tentative fragmentation pathway of the [M + HCOO^−^]^−^ ion for compound **47**. Figure S16: (a) TOF-MS/MS spectrum and (b) tentative fragmentation pathway of the theoretical [M−H]^−^ ion for compound **48**. Figure S17: (a) TOF-MS/MS spectrum and (b) proposed fragmentation pathway of the [M−H]^−^ ion for compound **49**.
