# Supplementary material for: Calendula arvensis (Vaill.) L.: A Systematic Plant Analysis of the Polar Extracts from Its Organs by UHPLC-HRMS
Source: Foods. 2022 Jan 18;11(3):247. doi: 10.3390/foods11030247 (PMC8834175; doi:10.3390/foods11030247)
Supplement: Supplementary file 1 [file foods-11-00247-s001.zip › foods-1543562-supplementary.pdf]

foods

Supplementary Materials

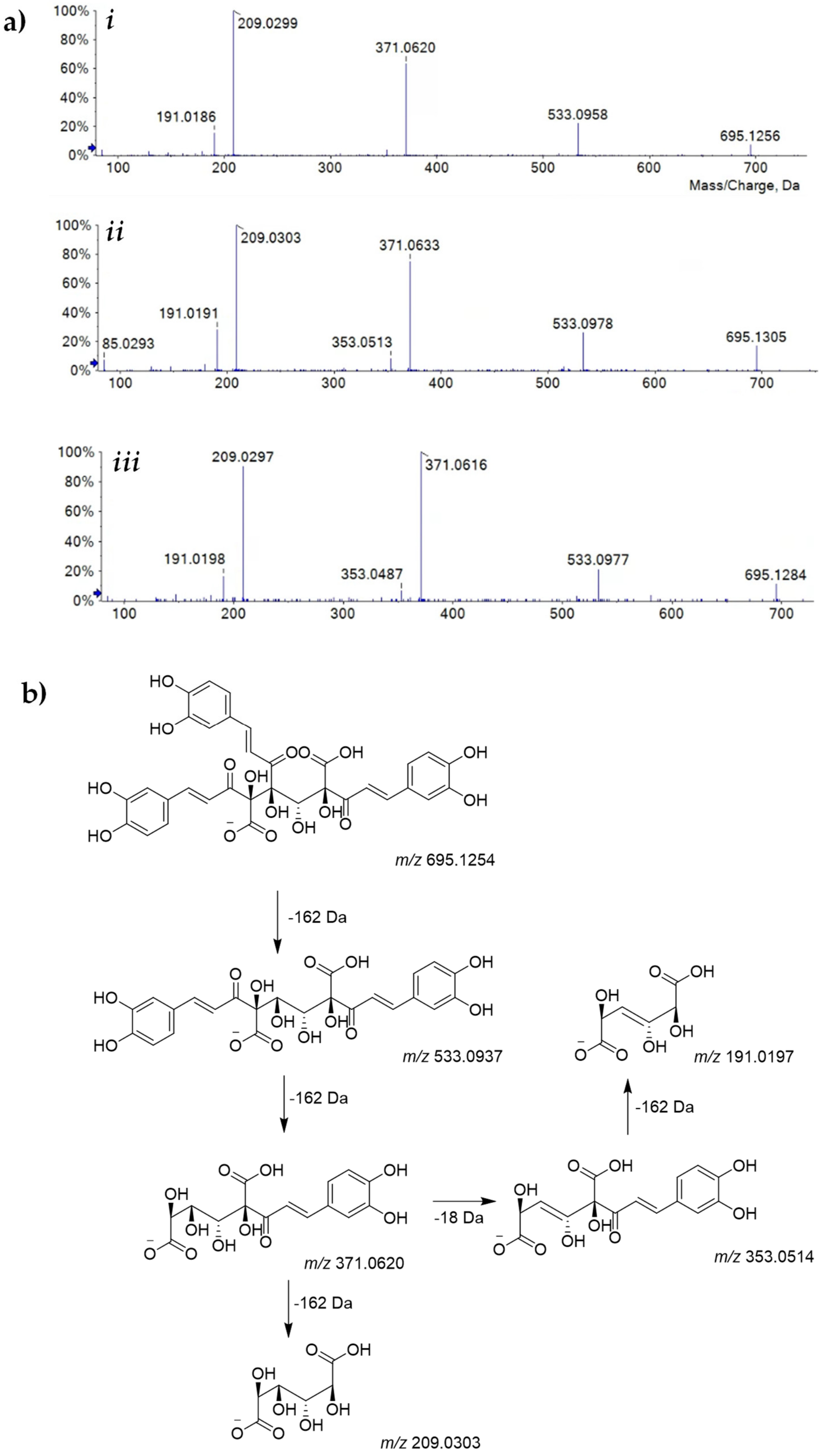

Figure S1. (**a**) TOF-MS/MS spectrum of the compounds (i) **30**, (ii) **31**, and (iii) **33**; and (**b**) tentative fragmentation pathway of the theoretical [M-H]- ion at *m/z* 695.1254.

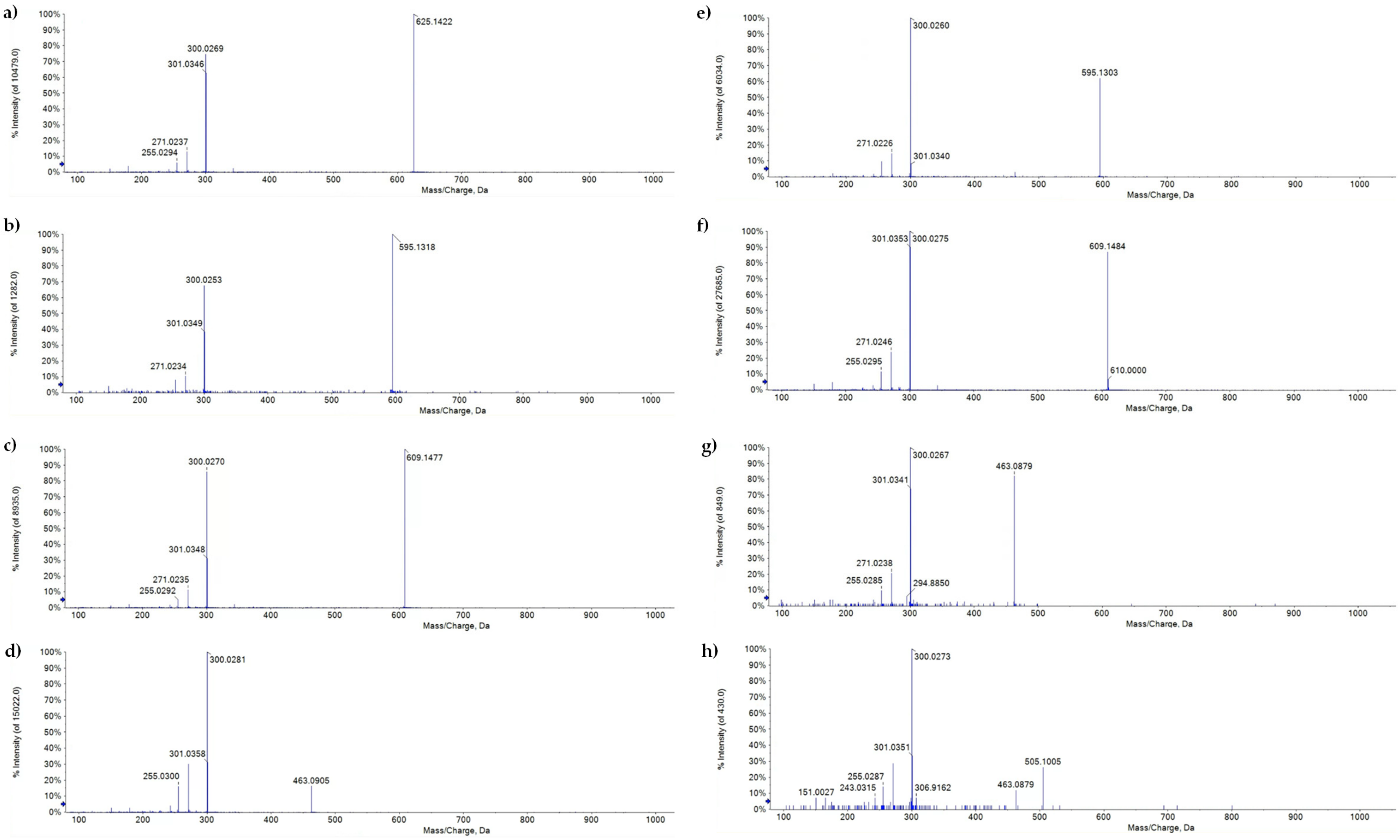

Figure S2. TOF-MS/MS spectrum of the quercetin glycosides from the floret alcoholic extract (a) **10**, (b) **11**, (c) **12**, (d) **13**, (e) **14**, (f) **15**, (g) **16** and (h) **22**. Neutral losses of 324.10 (**10** and **12**), 294.09 (**11** and **14**), 308.11 (**12** and **15**), 162.05 (**13** and **16**), and 204.06 (**22**) allowed the glyconic moiety to be recognized.

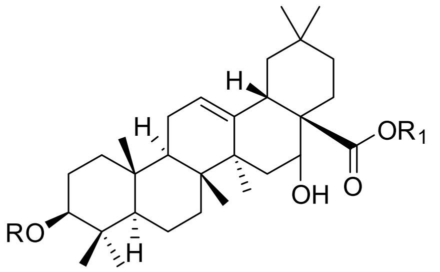

36 R = hex-hex-hex; R_1 = hex
37 R = hex-hexu; R_1 = hex
38 R = hex-hex; R_1 = hex
39 R = hexu; R_1 = hex
41 R = HMG-hex-hexu; R_1 = H
49 R = HMG-hexu; R_1 = H

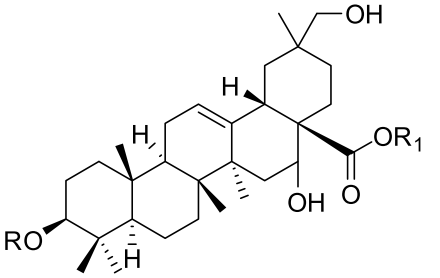

40 R = hexu; R_1 = hex

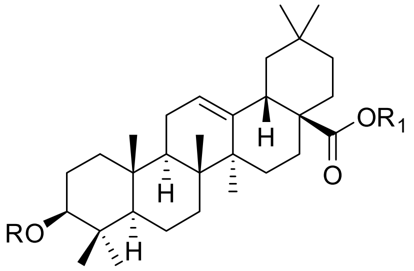

42 R = hex-hex-hex; R_1 = hex
43 R = hex-hexu; R_1 = hex
44 R = hex-hex; R_1 = hex
45 R = hex; R_1 = hex
46 R = hexu; R_1 = hex
47 R = hexu; R_1 = Achex
48 R = HMG-hexu; R_1 = hex

Figure S3. Chemical structures of triterpene saponins (**36** – **49**) tentatively identified in the *C. arvensis* alcoholic extracts. Hex = hexose; Hexu = hexuronic acid; Achex = acetylhexose; HMG = Hydroxymethylglutaric acid.

(a)

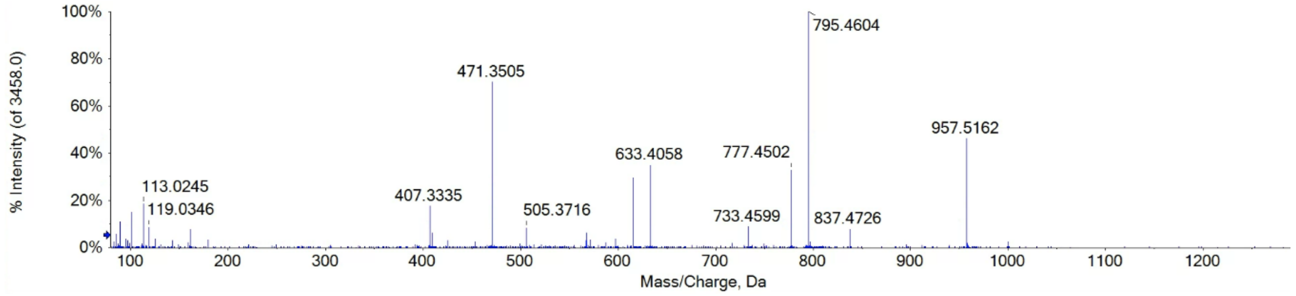

(b)

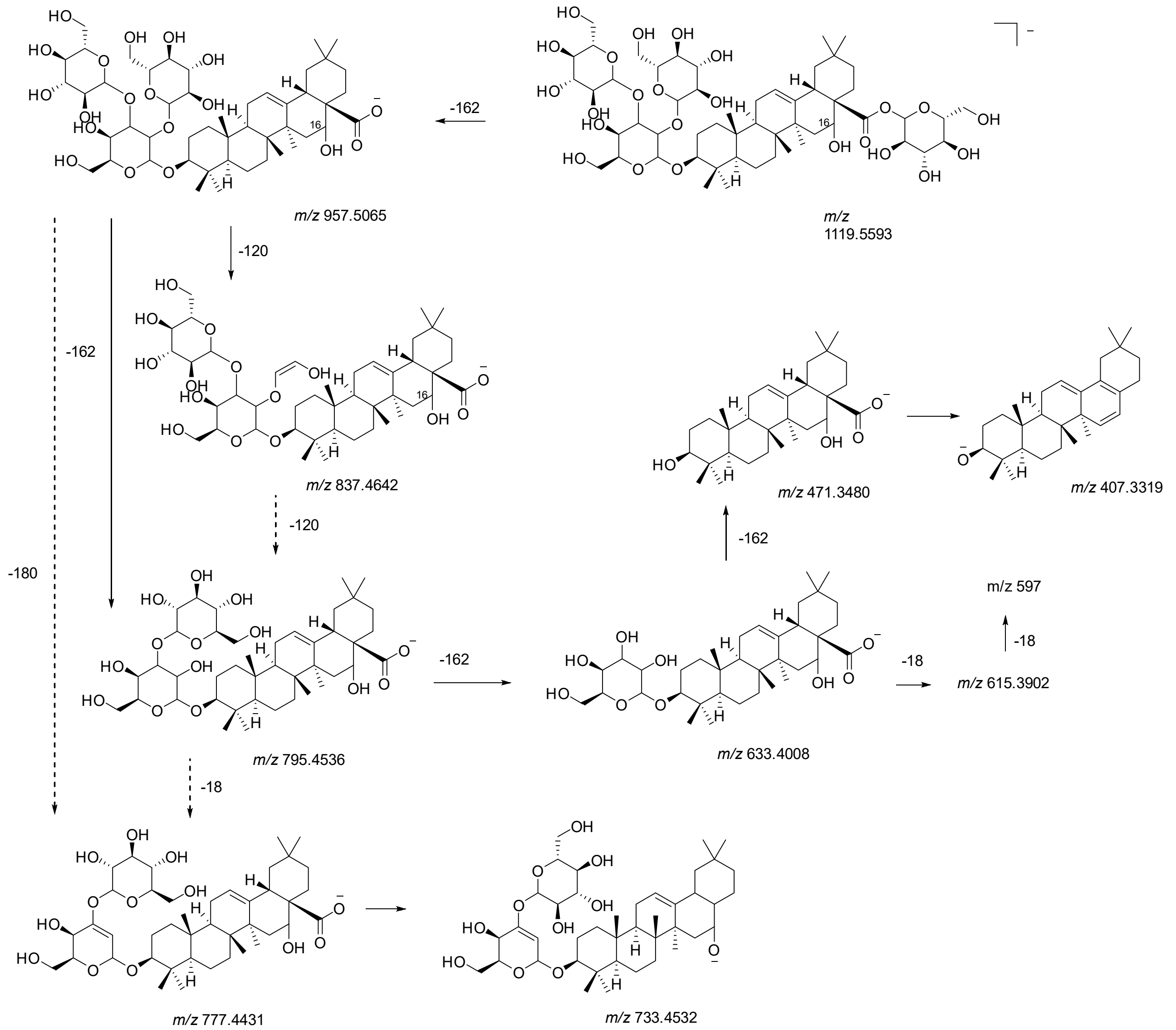

Figure S4. (**a**) TOF-MS/MS spectrum, and (**b**) tentative fragmentation pathway of the theoretical [M-H]- ion for compound **36**.

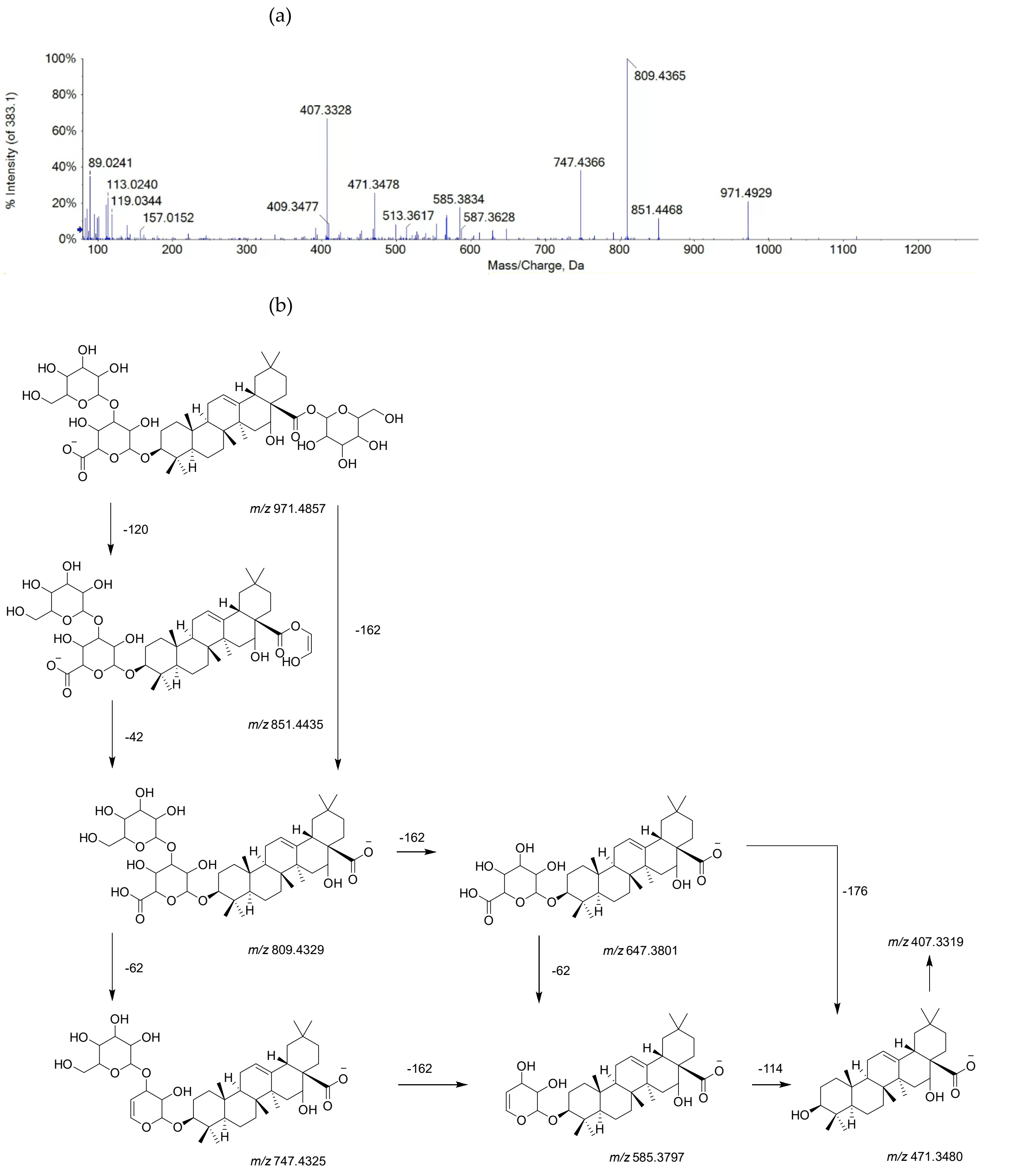

Figure S5. (**a**) TOF-MS/MS spectrum, and (**b**) tentative fragmentation pathway of the theoretical [M-H]- ion for compound **37**.

(a)

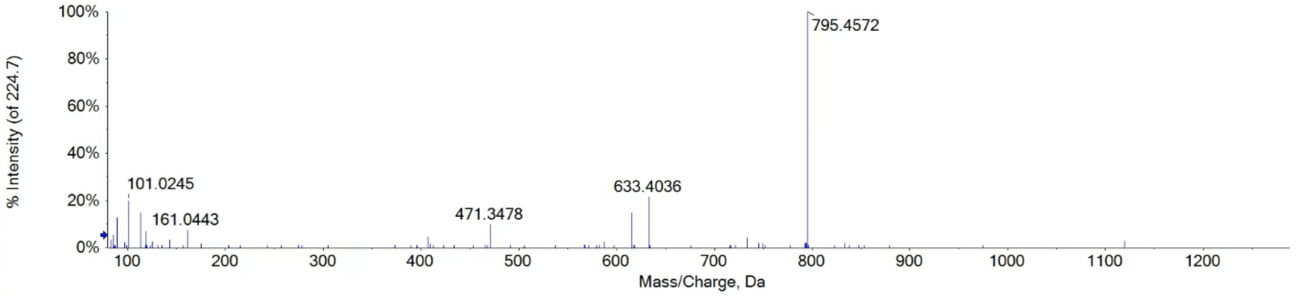

(b)

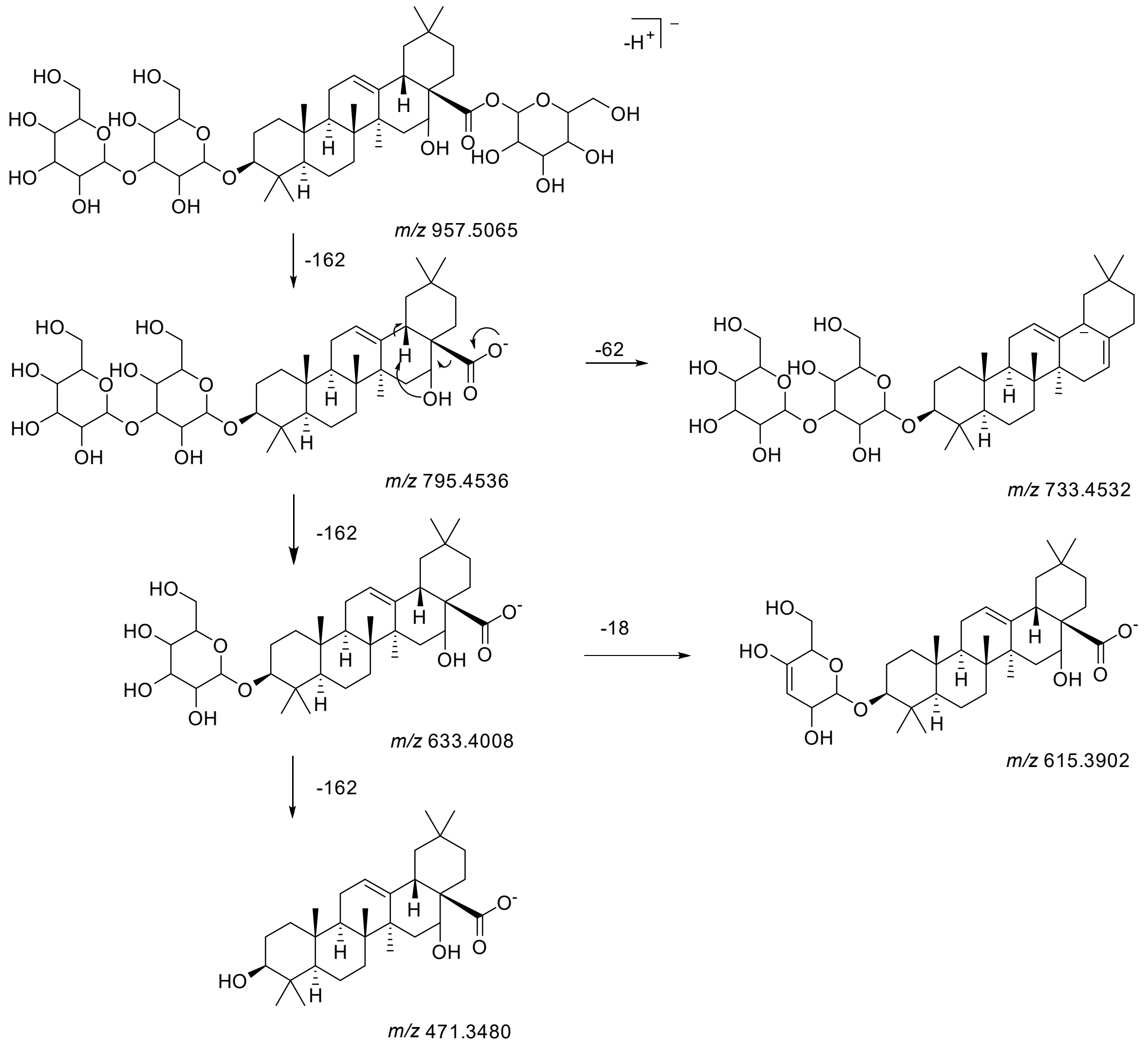

Figure S6. (**a**) TOF-MS/MS spectrum, and (**b**) tentative fragmentation pathway of the theoretical [M-H]- ion for compound **38.**

(a)

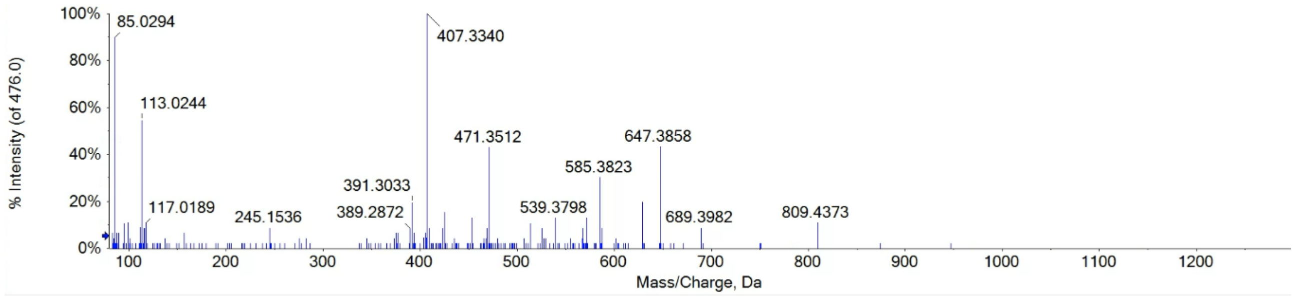

(b)

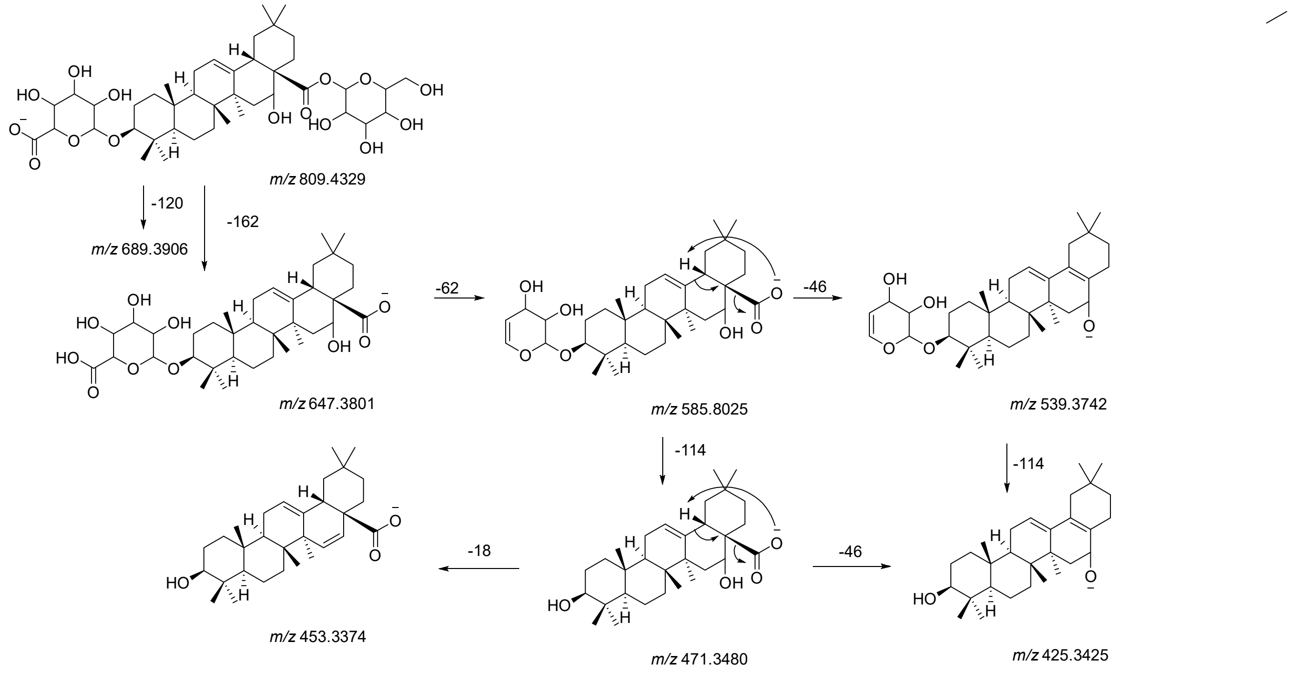

Figure S7. (**a**) TOF-MS/MS spectrum, and (**b**) tentative fragmentation pathway of the theoretical [M-H]⁻ ion for compound **39**.

(a)

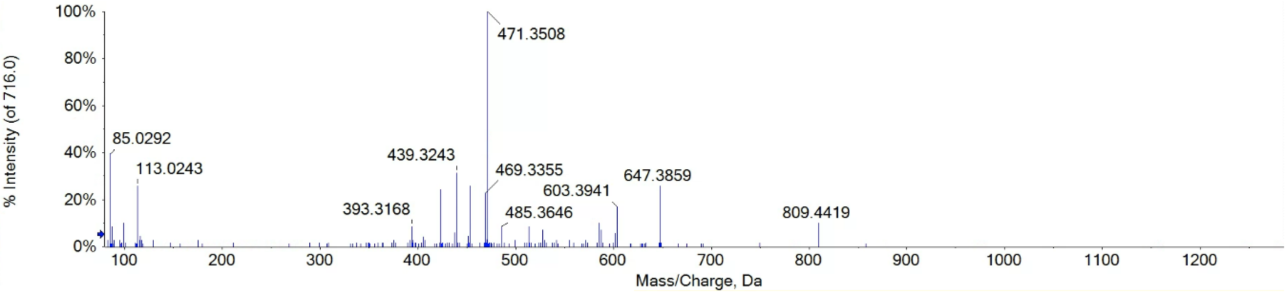

(b)

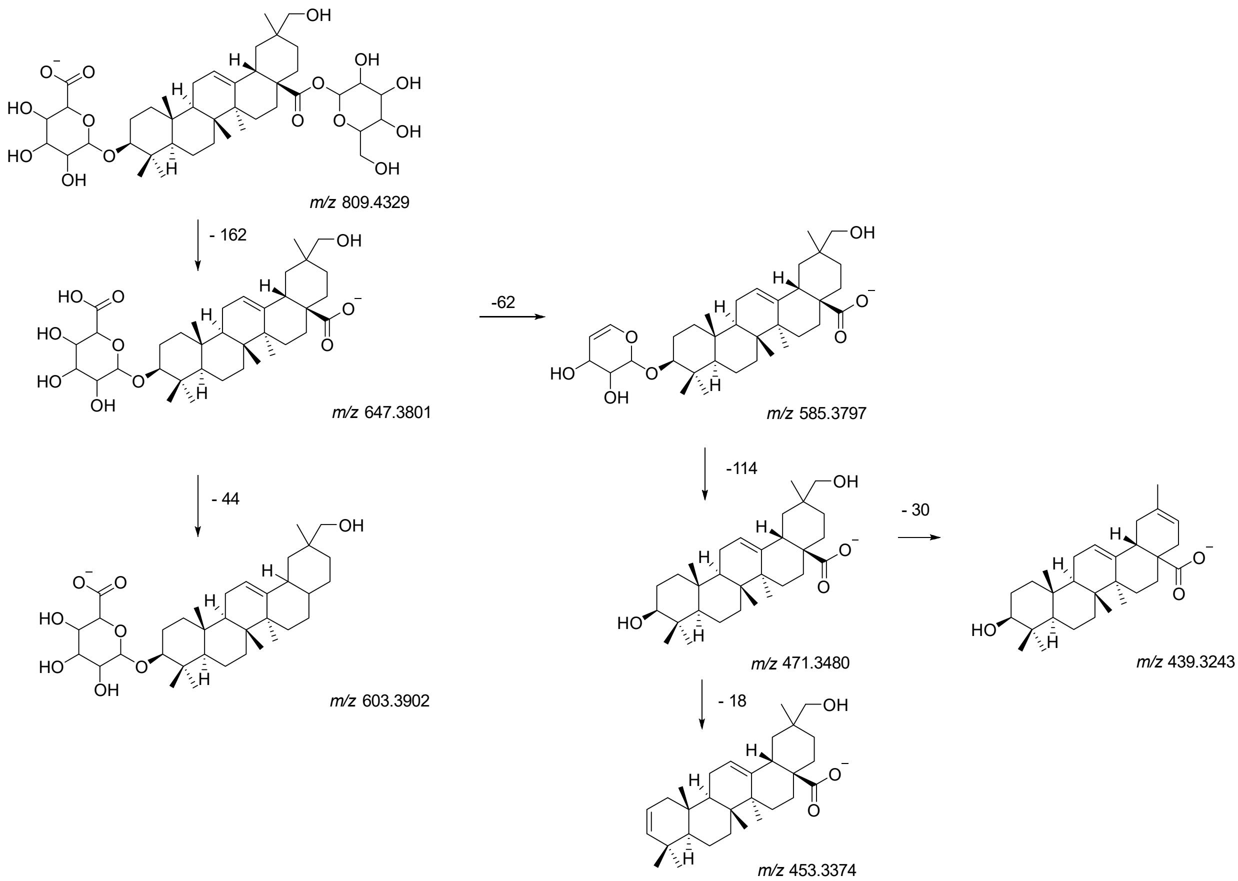

Figure S8. (**a**) TOF-MS/MS spectrum, and (**b**) tentative fragmentation pathway of the theoretical [M-H]- ion for compound **40**.

(a)

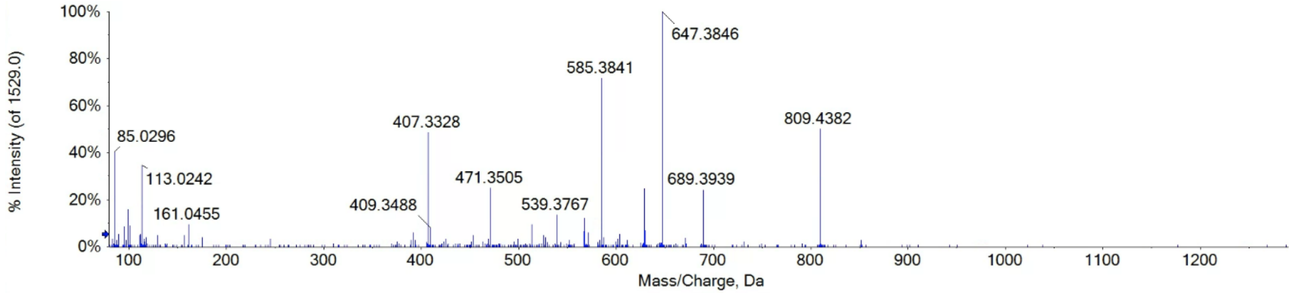

(b)

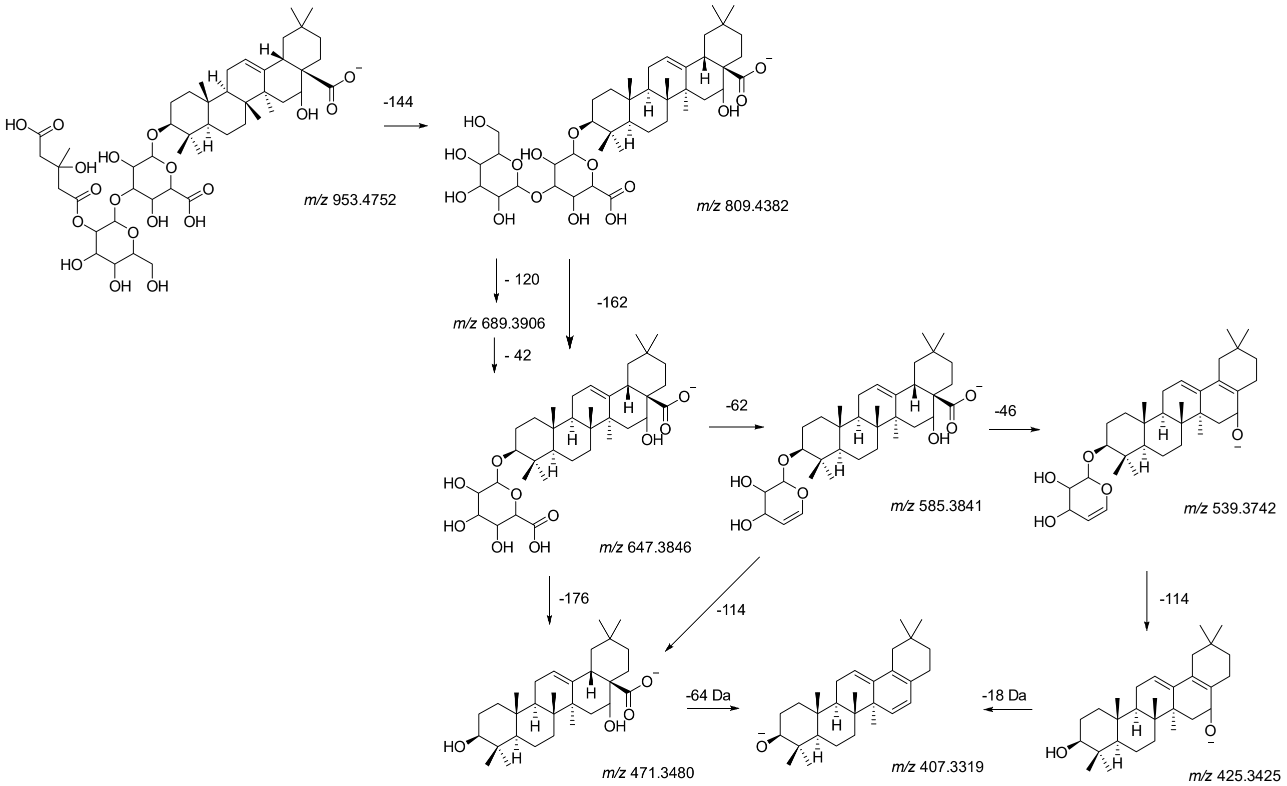

Figure S9. (**a**) TOF-MS/MS spectrum, and (**b**) tentative fragmentation pathway of the theoretical [M-H]⁻ ion for compound **41**.

(a)

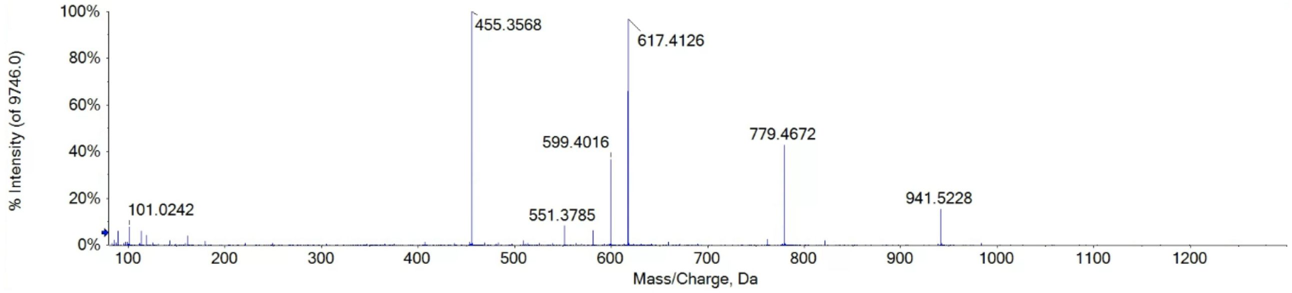

(b)

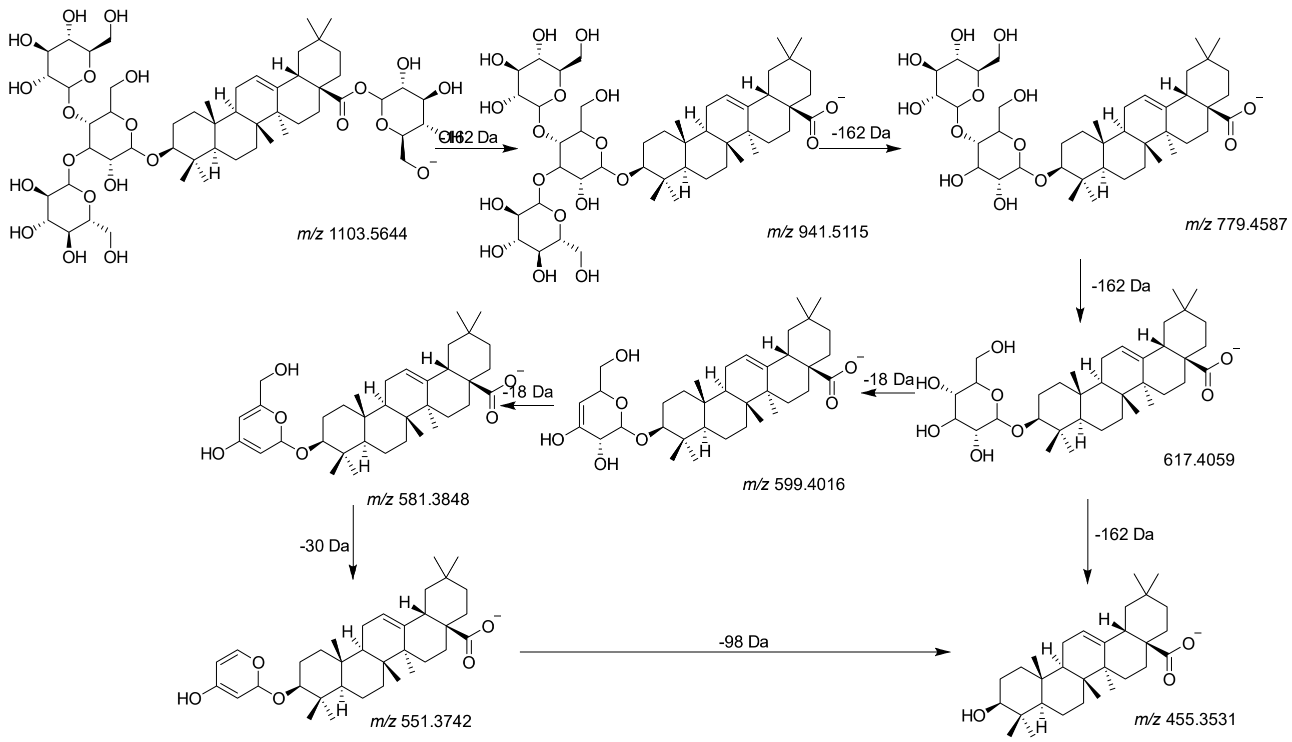

Figure S10. (**a**) TOF-MS/MS spectrum, and (**b**) tentative fragmentation pathway of the theoretical [M-H]$^-$ ion for compound **42**.

(a)

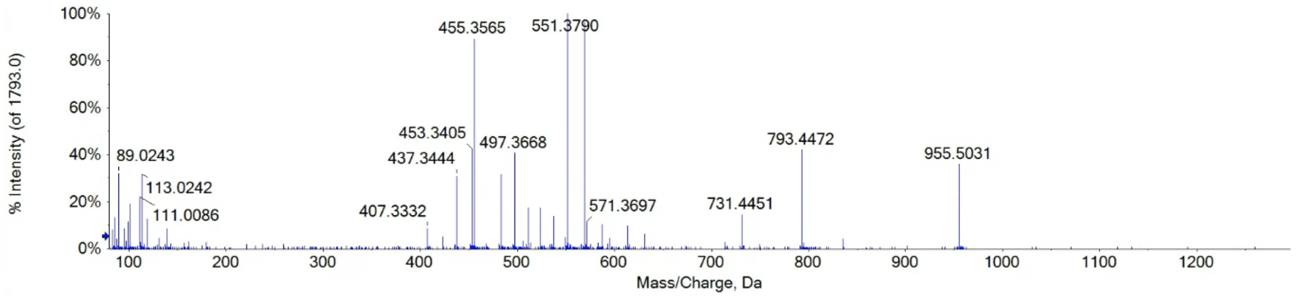

(b)

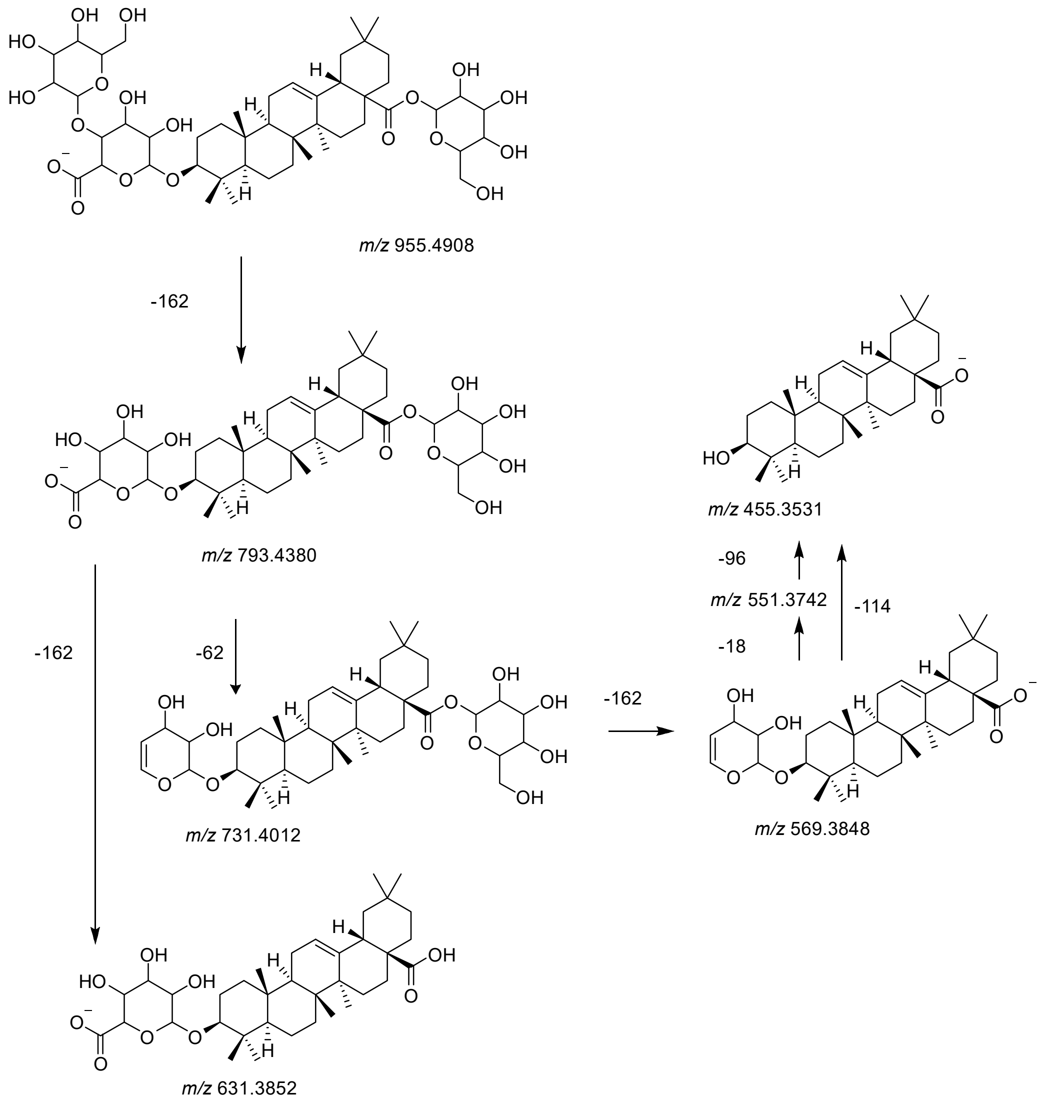

Figure S11. (**a**) TOF-MS/MS spectrum, and (**b**) tentative fragmentation pathway of the theoretical [M-H]- ion for compound **43**.

(a)

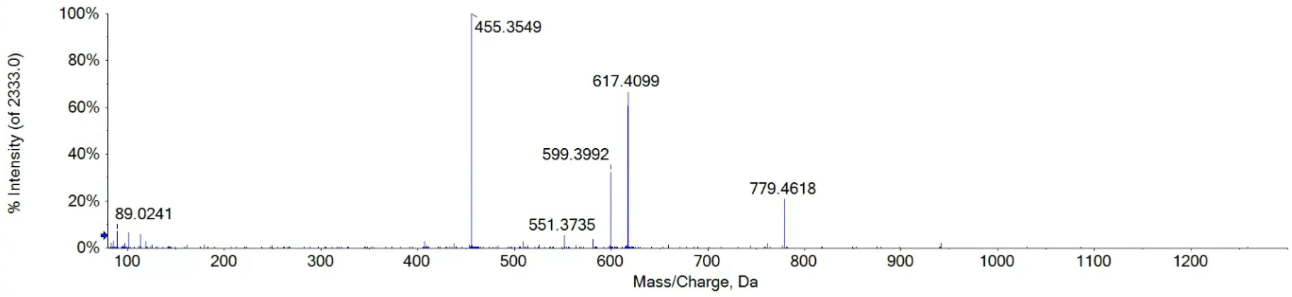

(b)

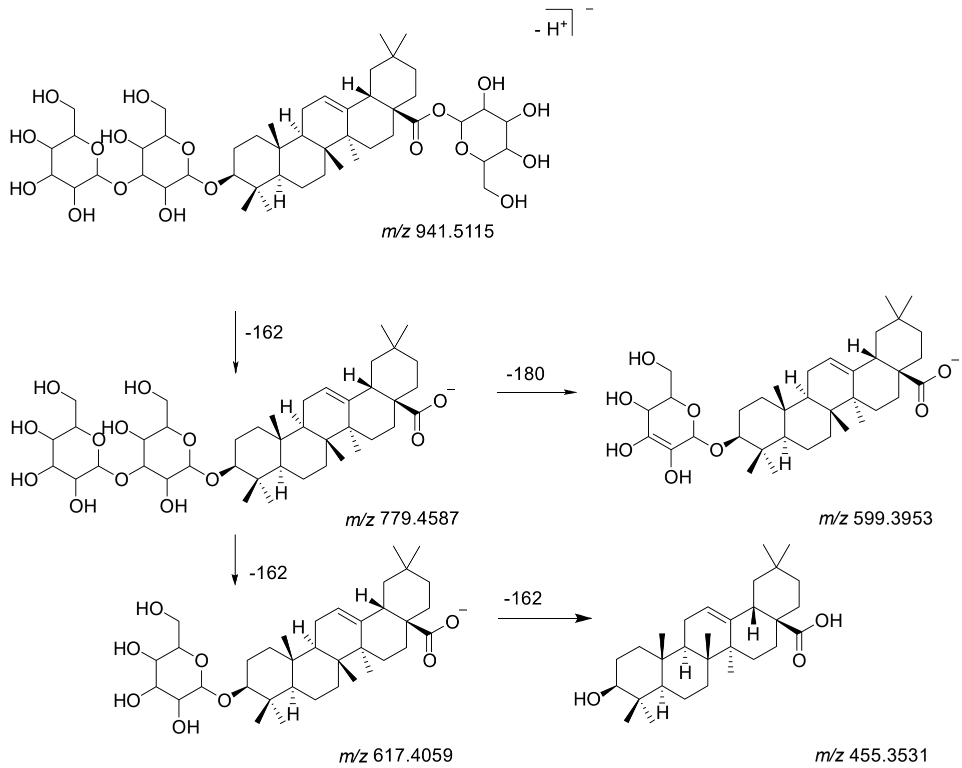

Figure S12. (**a**) TOF-MS/MS spectrum, and (**b**) tentative fragmentation pathway of the theoretical [M+HCOO⁻]⁻ ion for compound **44**.

(a)

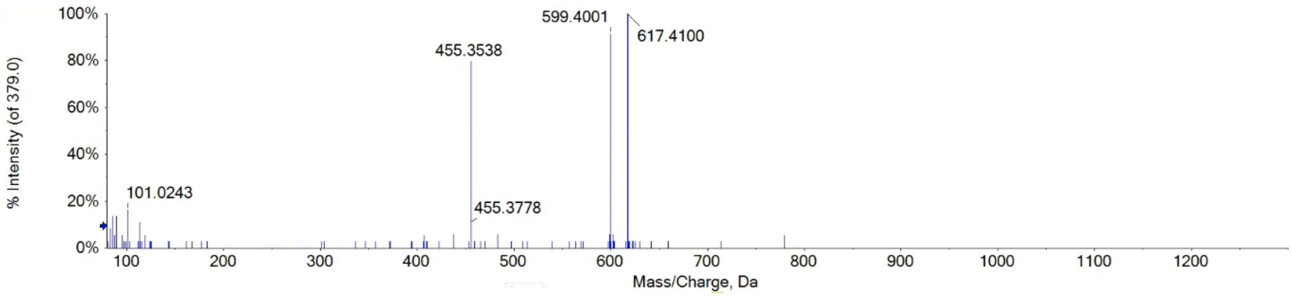

(b)

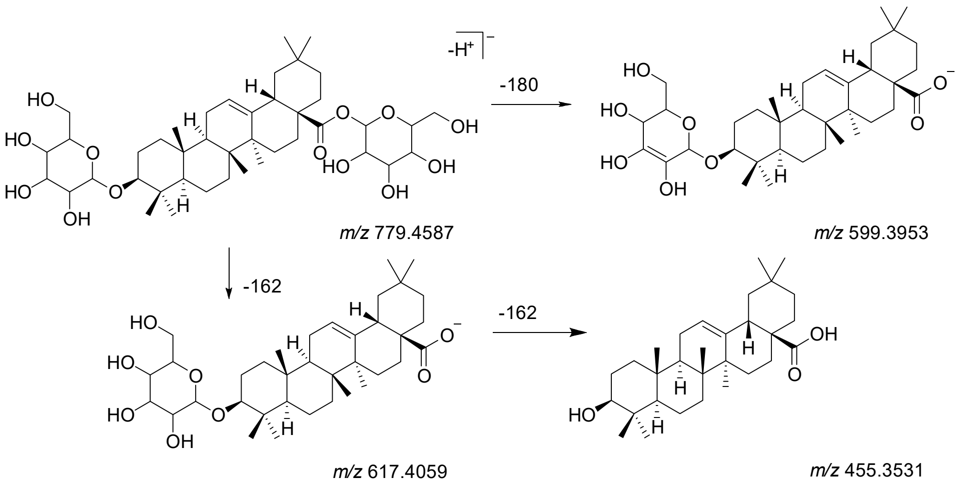

Figure S13. (**a**) TOF-MS/MS spectrum, and (**b**) tentative fragmentation pathway of the theoretical $[M+HCOO^{-}]^{-}$ ion for compound **45**. The theoretical *m*/*z* value is reported below each structure.

(a)

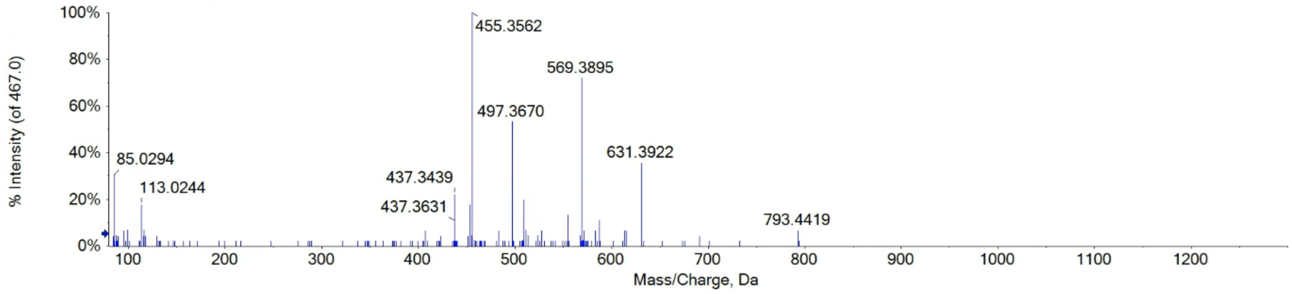

(b)

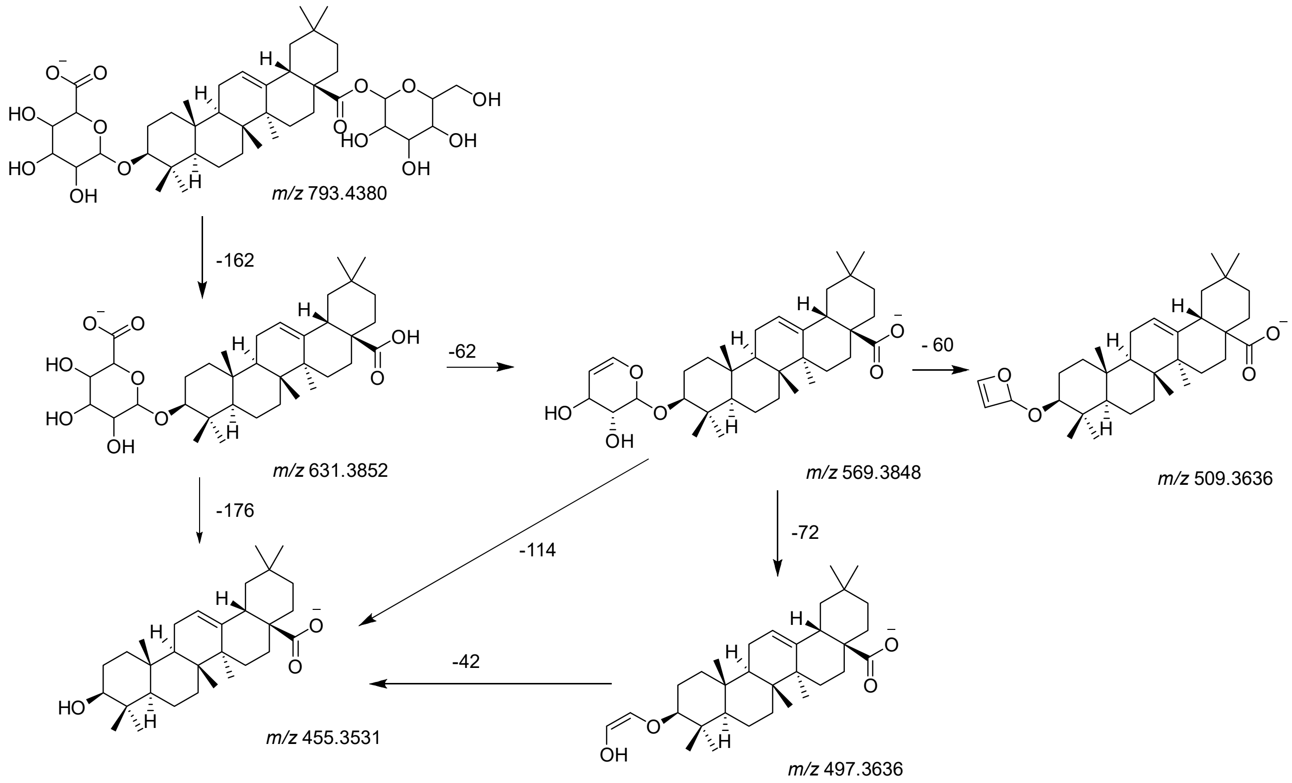

Figure S14. (**a**) TOF-MS/MS spectrum, and (**b**) tentative fragmentation pathway of the theoretical [M-H]- ion for compound **46**.

(a)

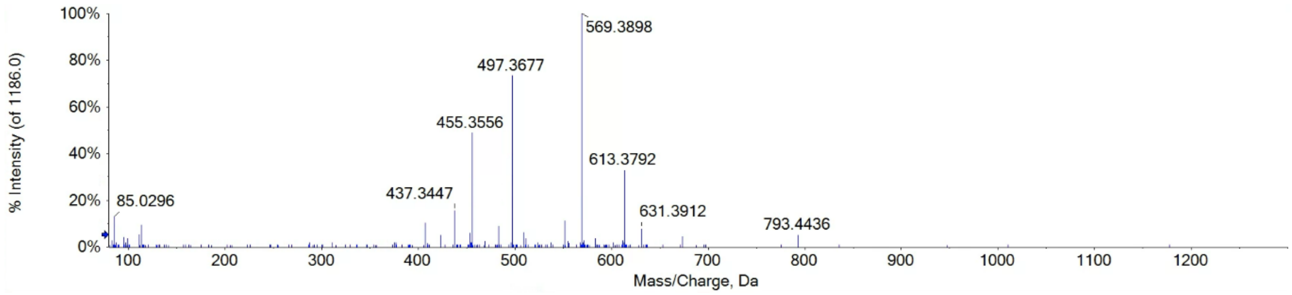

(b)

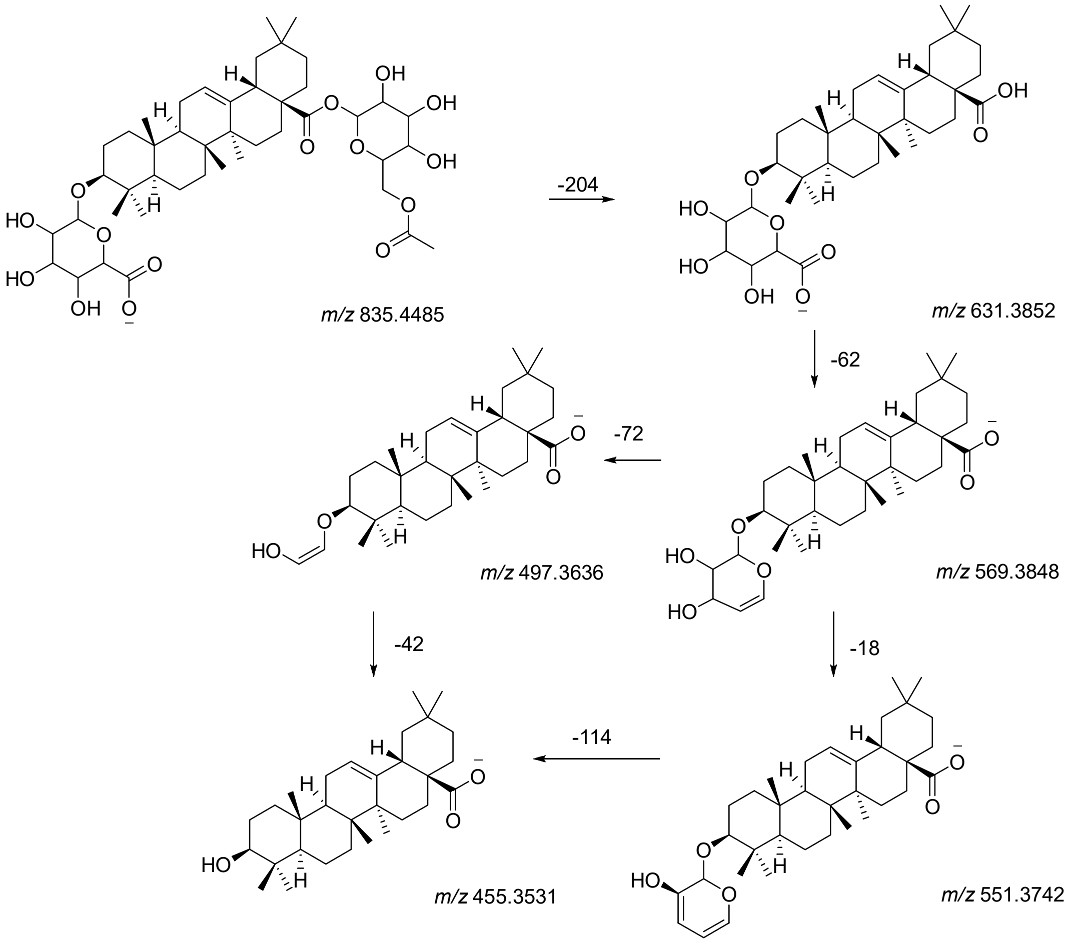

Figure S15. (**a**) TOF-MS/MS spectrum, and (**b**) tentative fragmentation pathway of the theoretical [M+HCOO⁻]⁻ ion for compound **47**.

(a)

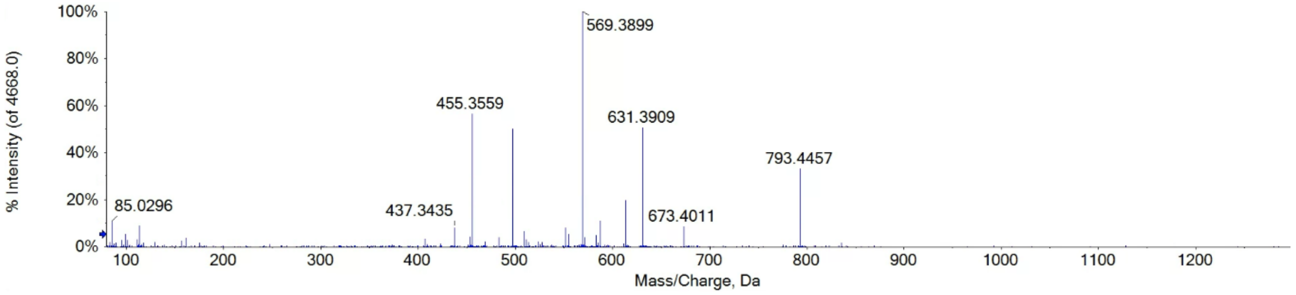

(b)

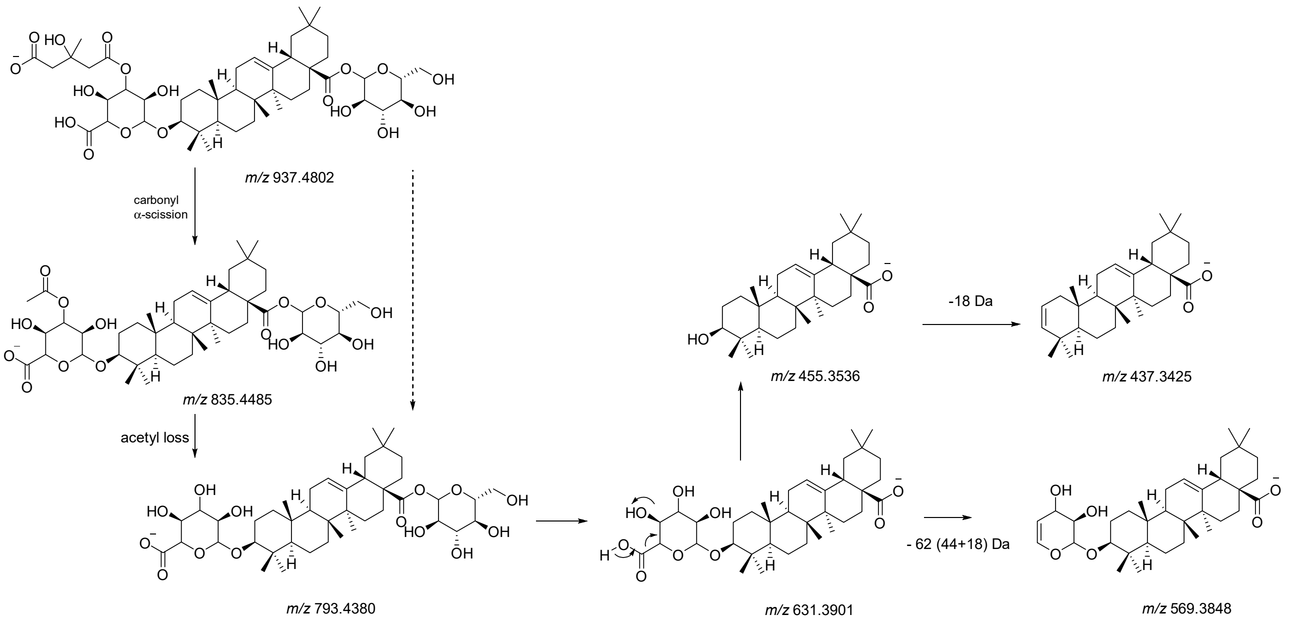

Figure S16. (**a**) TOF-MS/MS spectrum, and (**b**) tentative fragmentation pathway of the theoretical [M-H]$^-$ ion for compound **48**.

(a)

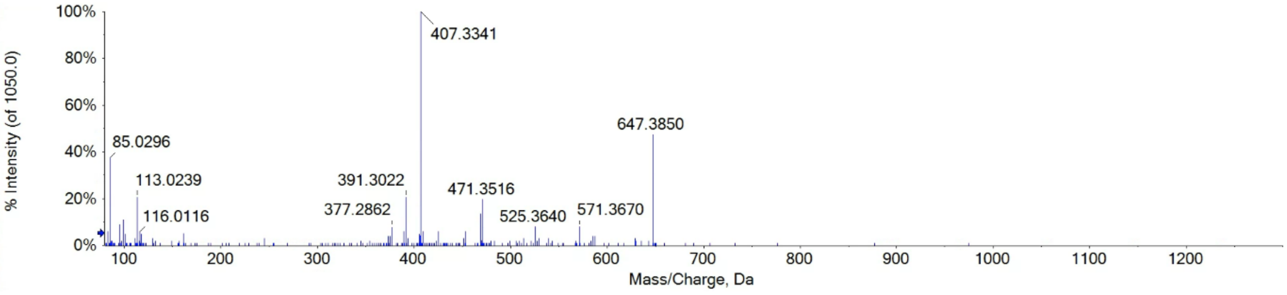

(b)

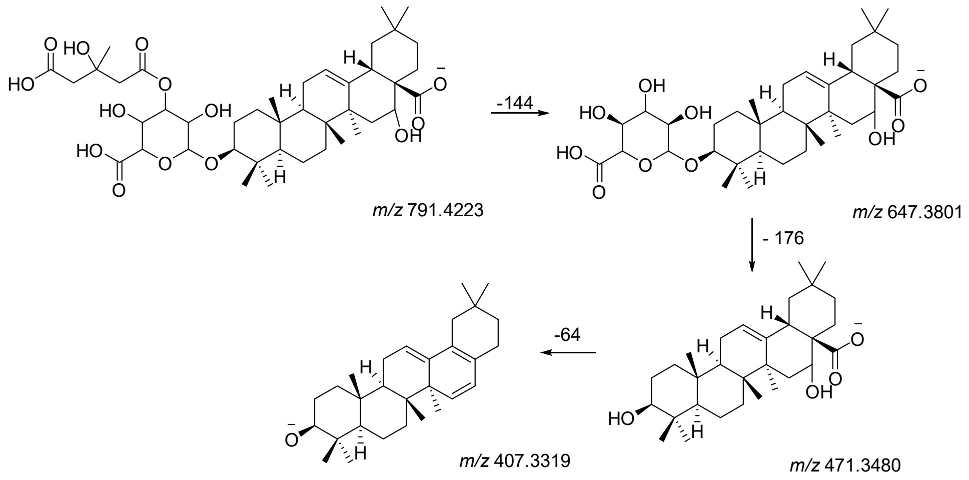

Figure S17. (**a**) TOF-MS/MS spectrum, and (**b**) tentative fragmentation pathway of the theoretical [M-H]$^{-}$ ion for compound **49**.
